# New insights into GATOR2-dependent interactions and its conformational changes in amino acid sensing

**DOI:** 10.1042/BSR20240038

**Published:** 2024-03-13

**Authors:** Can Yang, Xuan Sun, Geng Wu

**Affiliations:** State Key Laboratory of Microbial Metabolism, School of Life Sciences and Biotechnology, the Joint International Research Laboratory of Metabolic and Developmental Sciences MOE, Shanghai Jiao Tong University, Shanghai, China

**Keywords:** AlphaFold2-Multimer prediction, amino acid sensing, amino acid sensors, conformational change, GATOR2, mTORC1

## Abstract

Eukaryotic cells coordinate growth under different environmental conditions via mechanistic target of rapamycin complex 1 (mTORC1). In the amino-acid-sensing signalling pathway, the GATOR2 complex, containing five evolutionarily conserved subunits (WDR59, Mios, WDR24, Seh1L and Sec13), is required to regulate mTORC1 activity by interacting with upstream CASTOR1 (arginine sensor) and Sestrin2 (leucine sensor and downstream GATOR1 complex). GATOR2 complex utilizes β-propellers to engage with CASTOR1, Sestrin2 and GATOR1, removal of these β-propellers results in substantial loss of mTORC1 capacity. However, structural information regarding the interface between amino acid sensors and GATOR2 remains elusive. With the recent progress of the AI-based tool AlphaFold2 (AF2) for protein structure prediction, structural models were predicted for Sentrin2-WDR24-Seh1L and CASTOR1-Mios β-propeller. Furthermore, the effectiveness of relevant residues within the interface was examined using biochemical experiments combined with molecular dynamics (MD) simulations. Notably, fluorescence resonance energy transfer (FRET) analysis detected the structural transition of GATOR2 in response to amino acid signals, and the deletion of Mios β-propeller severely impeded that change at distinct arginine levels. These findings provide structural perspectives on the association between GATOR2 and amino acid sensors and can facilitate future research on structure determination and function.

## Introduction

mTORC1 regulates cell growth by sensing and integrating various environmental factors, including stress, oxygen, growth factors, energy and amino acids [1–5]. Rag guanosine triphosphatases (GTPases) recruit mTORC1 to the lysosomal membrane in the presence of abundant amino acids, and the nucleotide-loading state of Rag GTPases mediates mTORC1 activity [6–9]. Upstream of Rag GTPases, several proteins convey cytosolic amino acid availability to mTORC1. Among these, amino acid sensors, GATOR2 and GATOR1 forms a sequential regulatory hub [10–12]. The GATOR2 complex, composed of five subunits (WDR59, Mios, WDR24, Seh1L and Sec13), activates mTORC1 by acting as a mutual receptor for the leucine sensor Sestrin2 and the arginine sensor CASTOR1 [13,14]. The GATOR1 complex, which has three subunits (DEPDC5, NPRL2 and NPRL3), plays a key role in inhibiting mTORC1 activation by stimulating GTP hydrolysis of RagA in the GTPase-activating protein (GAP) mode, which is the functional mode of GATOR1 [12,15,16]. Amino acid sensors and GATOR2, GATOR2 and GATOR1 act as antagonistic regulators of mTORC1 through direct interactions with each other. Specifically, when amino acids are abundant, Sestrin2 and CASTOR1 are in an amino acid-bound state and dissociate from GATOR2, allowing GATOR2 to inhibit GATOR1. As a result, the GTP-loaded state of Rag GTPase leads to the activation of mTORC1. In contrast, these two amino acid sensors inhibit mTORC1 during nutrient deprivation by interacting with GATOR2 and inhibiting the function of GATOR2.

Three of the five GATOR2 subunits (WDR59, Mios and WDR24) share a similar architecture, which possesses an N-terminal β-propeller (Nβ), α-solenoid domain and C-terminal RING domain. Both Seh1L and Sec13 carry six-bladed β-propellers [17]. A previous cryo-EM model of GATOR2 established the intra-molecular interactions between five subunits [18]. It has been reported that the three large subunits (WDR59, Mios and WDR24) form a scaffold via heterodimeric CTD^RING^-CTD^RING^ junctions and the α-solenoid interactions. Each large subunit donates an extra β-blade to complete the open six-bladed β-propellers of Seh1L or Sec13. GATOR2 uses distinct binding strategies for these two amino acid sensors. For Sestrin2, the GATOR2-binding interface requires WDR24 Nβ (residues 1–327) and Seh1L with one β-blade donation from WDR24 [18,19]. Recent research has validated the interaction between RING domain of WDR24 and Sestrin2 and implied that the dissociation of Sestrin2 may trigger a conformational change in RING domain interface of Mios and WDR24 [20]. Mios Nβ (residues 1–352) alone is sufficient to associate with the dimeric CASTOR1 [18,21]. However, there is no structural information about the interactions between amino acid sensors and GATOR2, nor is it understood whether the interaction contributes to the conformational change in GATOR2.

There are three main approaches to determining the atomic resolution of proteins or protein complexes. Structures that can be determined by NMR are mostly small proteins or peptides [22]. X-ray crystallography requires efforts to screen constructs and purify abundant protein samples to culture protein crystals using expensive reagent kits [23,24]. Despite its clear advantages, including the accessibility of large protein complexes using cryo-electron microscopy (EM) to understand structural mechanisms, obtaining high-resolution models can be a prohibitive and time-consuming endeavour in terms of protein sample preparation and data processing [25,26]. Critical information about the 3D structure is embedded in the sequence, which makes it possible to predict the protein structure computationally [27,28]. In 2021, the AF2 software initiated a funsdamental change, a deep-learning approach developed by DeepMind, which showed high accuracy in predicting protein structures [29]. The released AF2-multimer program has become a robust tool for protein complex predictions and is shared by the broad scientific community [30]. For example, it assists in defining the different multimeric states of most proteins in solution, accurately predicting canonical and noncanonical ATG8 interacting motifs, and indicating the potential binding mode for multi-spanning membrane proteins [31–33].

In the present study, we applied the AF2-multimer algorithm to build models of Sestrin2-WDR24-Seh1L and CASTOR1-Mios Nβ. These models led us to identify the conserved residues within the interfaces between amino acid sensors and Nβ region of Mios and WDR24. Furthermore, the WDR24 binding sites in Sestrin2 are crucial for maintaining mTORC1 capacity. By combining normal mode analysis and FRET measurements, we discovered that GATOR2 underwent a conformational switch induced by arginine and leucine signals. Molecular dynamic (MD) simulations were performed to understand the mutagenic impact on the interactions between WDR24 and Sestrin2, and the conformational changes in GATOR2. In summary, the present study sheds light on the basis for GATOR2-amino acid sensor interactions and reveals the conformational changes of GATOR2 in response to arginine and leucine.

## Results

### AF2-multimer predicts the model of Sestrin2-WDR24-Seh1L complex with high accuracy

**Figure 1:**
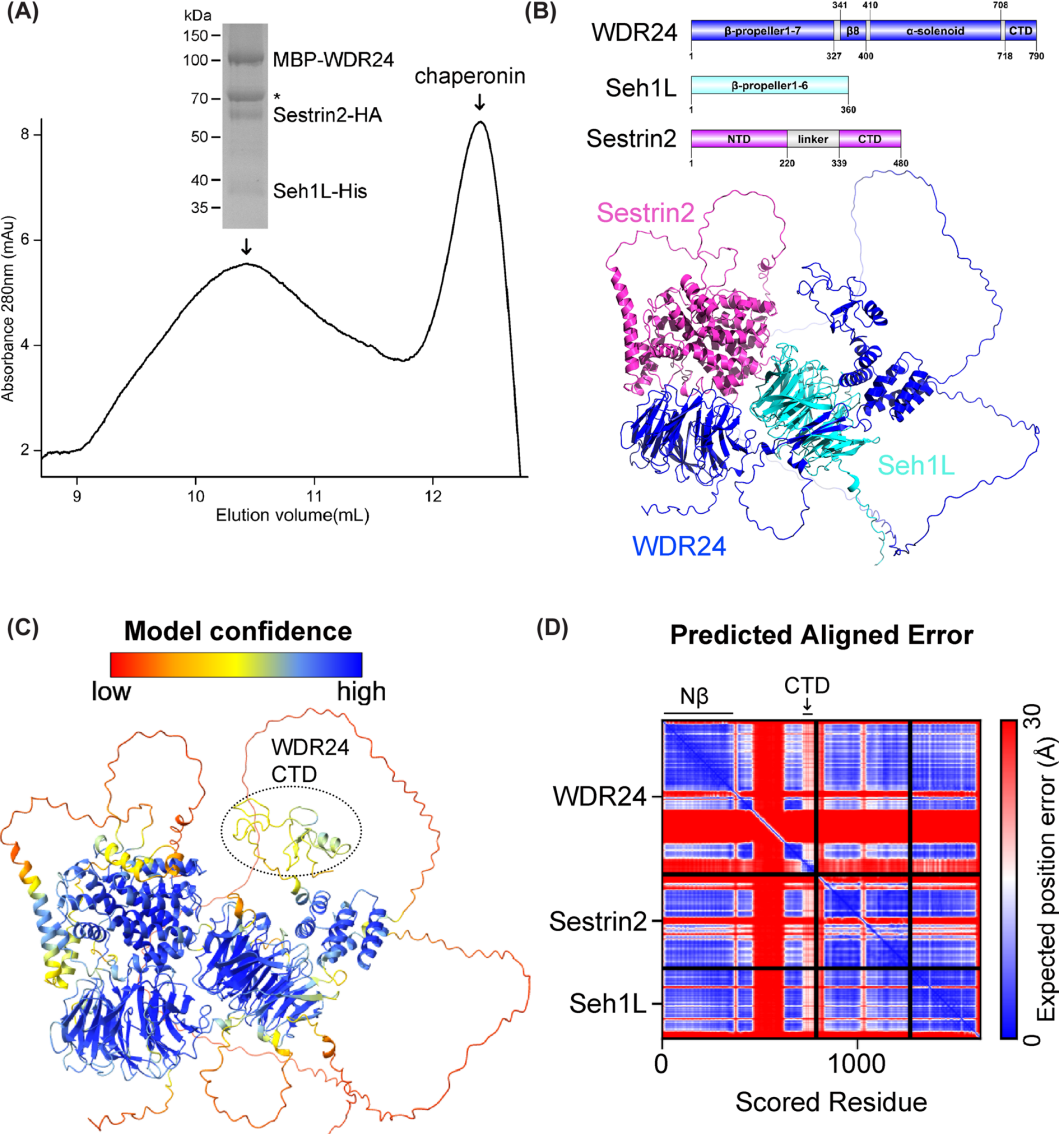
The structure of Sestrin2-WDR24-Seh1L complex is predicted by AF2-multimer (**A**) Size-exclusion chromatography profile for MBP-WDR24, Sestrin2-HA and Seh1L-His with the indicated coomassie blue-stained SDS-PAGE gel of purified Sestrin2-WDR24-Seh1L (the protein chaperon, HSP70, indicated with asterisk). (**B**) Cartoon representation of AF2-multimer prediction for Sestrin2 (coloured in red), WDR24 (coloured in slate) and Seh1L (coloured in cyan). Domain organization of each component depicted by DOG2.0 software with numbers denoting the regions of the indicated domains. (**C**) Cartoon representation of the complex model in (**B**), colour-coded by the pLDDT values. (**D**) The Predicted Aligned Error of the model in (B) with the high confidence of the contract regions of WDR24 Nβ and Sestrin2. Above the plot, the corresponding domains are indicated. Within the plot, the black lines denote the area of individual protein of the complex.

As previously mentioned, two subunits of GATOR2, WDR24 and Seh1L are involved in the interaction with Sestrin2 in an *in vivo* assay (immunoprecipitation) [18,19]. To investigate the interactions between Sestrin2, WDR24 and Seh1L in the mammalian expression system, the three components were co-expressed in Expi293F cells and purified by affinity chromatography with the following size-exclusion chromatography (Figure 1A). This indicates that WDR24 collaborates with Seh1L to enable direct physical interaction between GATOR2 and Sestrin2.

Probing into the protein complex of Sestrin2, WDR24 and Seh1L, AF2-multimer prediction was conducted on the full-length protein sequences as three separate chains. The resulting five models are ranked according to the pLDDT (predicted local distance difference test), predicted aligned error (PAE) and interface predicted TM (ipTM) score. Notably, five models possess the same modular arrangements (Figure 1B and Supplementary Figure S1A). Per-residue confidence for each model was estimated by the pLDDT values ranging from 0 to 100 (Supplementary Figure S1B), and detailed pLDDT values from the top-ranked model was depicted (Figure 1C). Most residues in the predicted model had very high confidence scores (pLDDT >90, Figure 1C). One flexible linker (residues 459–625) of WDR24 in AF2 predicted model showed low accuracy (35 < pLDDT < 60), the density of the same linker (residues 459–625) between WDR24 Nβ and C-terminal domain (CTD; residues 718–790) is missing in cryo-EM structure of GATOR2 [18], suggesting that this region is disordered in nature. AF2 cannot accurately predict disordered or flexible regions, it is not unexpected that WDR24 CTD, consisting mostly of irregular structures, had lower pLDDT values ranging from 50 to 80 in the predicted model (Figure 1C). Although the RING domain (residues 742–787) of WDR24 was reported to have critical function for its association with Sestrin2 under leucine deprivation [20], the resulting predictions showed no direct contact between that the RING domain and Sestrin2 (Figure 1C,D), suggesting that the linker between the CTD and α-Solenoid region is flexible allowing the RING domain to exploit other possible orientations to optimise its contact with Sestrin2.

Overall, AF2-multimer provides structural models of Sestrin2-WDR24-Seh1L with consistent shape complementarity for further analysis in the present study.

### Mutating the conserved residues affect the interaction between Sestrin2 and WDR24

To experimentally verify the AF2 prediction and the proposed interfaces, we first analysed the highly confident interfaces between Sestrin2 and two β-propellers, WDR24 Nβ and Seh1L (Figure 1B). Using COCOMAPS online to generate contact maps with a threshold distance of interacting residues in 5 Å [34], the interactions of WDR24 Nβ and Sestrin2 were mainly in the CTD (residues 339–480) of Sestrin2, while the N-terminal domain (NTD; residues 66–220) of Sestrin2 exclusively binds to Seh1L (Supplementary Figure S2A). Previous mutagenesis studies proved that residue Ser190 in the NTD of Sestrin2, locating close to Asp111 and Leu115 of Seh1L in predicted models, is essential to bind GATOR2 [11].

**Figure 2:**
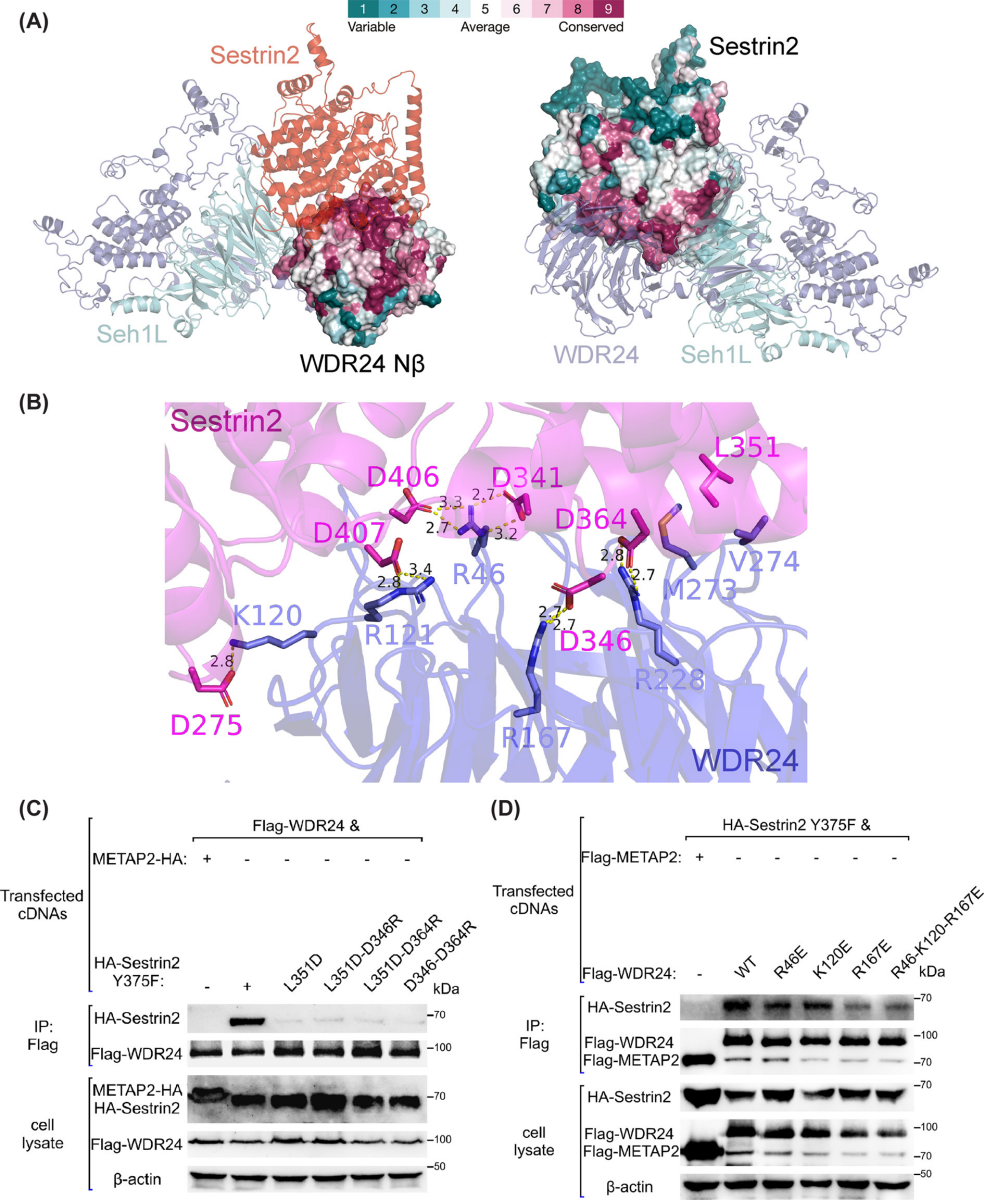
Assessment of the interaction interface between Sestrin2 and WDR24 Nβ (**A**) ConSurf analyses for WDR24 β-propellor (left) and Sestrin2 (right) as shown by surface. (**B**) The entire binding interface between Sestrin2 and WDR24 Nβ. Hydrogen bonds are shown as yellow dotted lines with indicated distances. (**C**) The binding of METAP2 (as a negative control), Sestrin2^Y375F^, and different Sestrin2^Y375F^-based mutants to WDR24 was assessed via anti-Flag co-IPs. (**D**) The binding of METAP2, WDR24 and different WDR24 mutants to Sestrin2^Y375F^ was evaluated by anti-Flag co-IPs. The results in (**C,D**) are representative of three independent experiments.

Concerning the interface between WDR24 Nβ and Sestrin2, evolutionary conservation was determined using the ConSurf web server [35,36], and the interaction interface was found to be highly conserved (Figure 2A). These interface interactions include the DD motif (Asp406 and Asp407) of Sestrin2, which was identified as required for Sestrin2–GATOR2 interaction and mTORC1 inhibition [14,37], forming hydrogen bonds with two arginine residues of WDR24 (Arg46 and Arg121, Figure 2B). Further, we tested the contributions of previously undiscovered residues within the interface including Asp346, Leu351, and Asp364 of Sestrin2 and Arg46, Lys120, and Arg167 of WDR24 Nβ (Figure 2B–D). These individual residues are conserved across biological kingdoms (Supplementary Figures S2B and S3). The flex dd*G* method was used to generate ΔΔ*G* values for binding free energies upon saturation mutations (Supplementary Figure S2C). Based on the destabilising effects shown in the matrixes of ΔΔ*G* calculation, we performed site-directed mutagenesis to verify the importance of these residues via *in vivo* anti-Flag co-IP experiments. Sestrin2 mutation Y375F was used in each co-IP experiment to abolish the interaction with leucine without affecting the binding of GATOR2 as previously demonstrated [14]. We first examined two single-site mutants of Sestrin2, D346R and D364R, and found that their contributions the interaction between Sestrin2 and WDR24 was very limited (data not shown). However, L351D mutant significantly abolished this interaction (Figure 2C). Furthermore, Sestrin2 double mutants (L351D-D346R, L351D-D364R and D346-D364R) also considerably inhibited complex formation (Figure 2C). The single-site mutants of WDR24, including R46E, K120E and R167E located in the Nβ of WDR24, results showed decreased binding affinity compared with wild-type WDR24 (Figure 2D). MD simulation was then used to gain insights into the contribution of the aforementioned residues. The overall structure of WDR24-Sestrin2 complex showed high stability in conventional MD simulation, and the pairwise WDR24-Sestrin2 interface residues remained unchanged except for Lys120 of WDR24. In the representative structures of all simulated trajectories, Lys120 of WDR24 showed two alternative conformations by forming a hydrogen bond with either Glu118 from WDR24 or Glu270 from Sestrin2 (Supplementary Figure S2D), which is further illustrated by the distance distribution in the free energy landscape (Supplementary Figure S2E). These computational data suggest a weak contribution of K120 to the binding of Sestrin2.

### Key interface residue mutations compromise mTORC1 sensitivity to leucine

**Figure 3:**
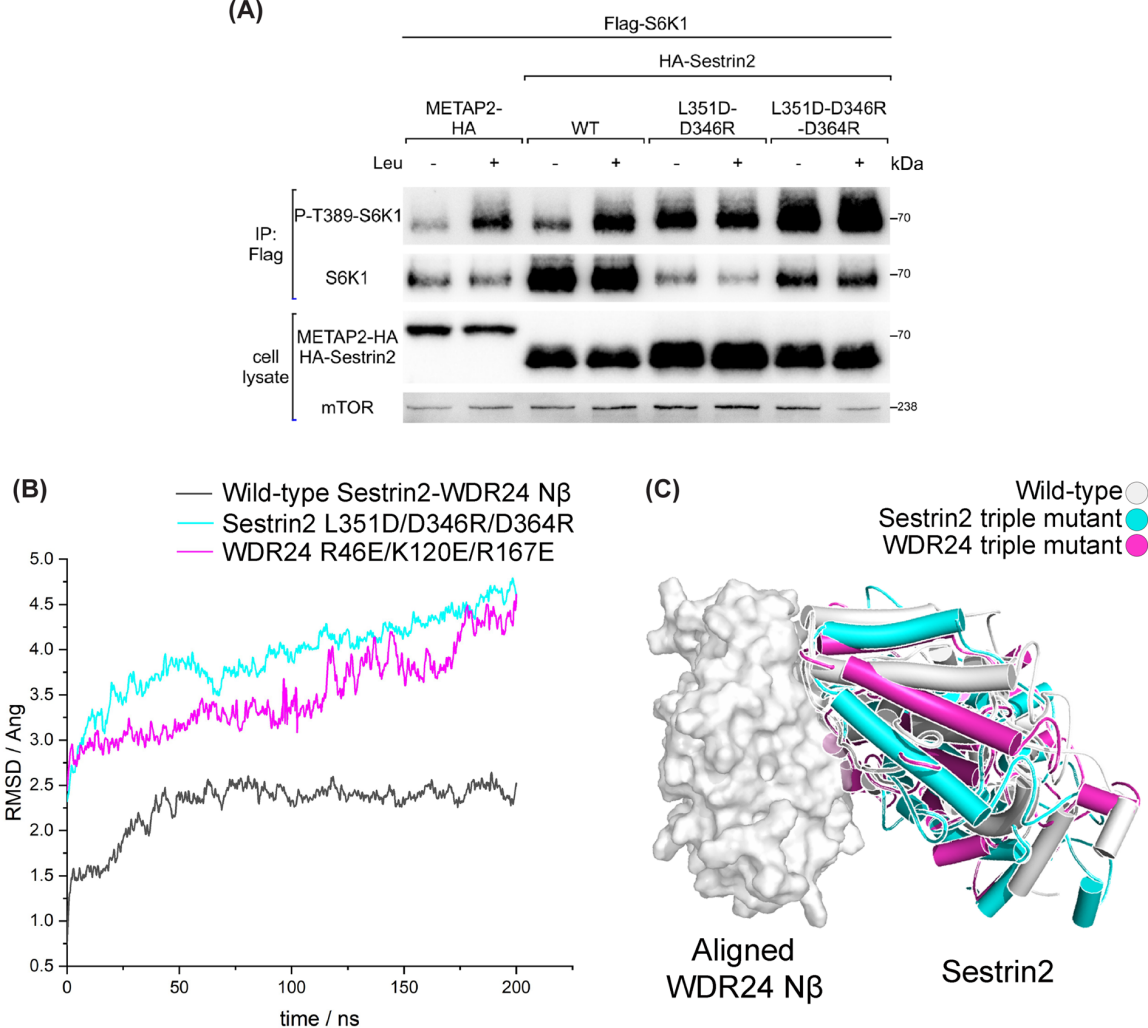
Sestrin2 mutants impair mTORC1 sensitivity to leucine (**A**) Effect of Sestrin2 mutants on the sensitivity of mTORC1 to leucine. HEK-293T cells are transiently transfected with Flag-S6K1 and the indicated Sestrin2 constructs. Anti-Flag IPs were prepared and analysed by immunoblotting. (**B**) Backbone rmsd of Sestrin2/WDR24 mutants relative to wild-type structure with the alignment of WDR24 β-propeller in three sets of MD runs. Triple mutation on WDR24 (coloured in magenta); triple mutation on Sestrin2 (coloured in cyan); wild-type structure (coloured in dark grey). (**C**) Aligned models of Sestrin2 (shown as cartoon) and WDR24 β-propeller (shown as surface and coloured in grey) complex generated from MD runs as described in (B).

To test the effect of Sestrin2 CTD mutants on mTORC1 signalling *in vivo*, WDR24 binding-deficient mutants of Sestrin2 were transiently overexpressed in HEK-293T cells. S6K1 was used as a direct readout of mTOR activation. In contrast with wild-type Sestrin2, both L351D-D346R and L351D-D364R-D364R mutants largely inhibit the sensitivity of mTORC1 in response to leucine signal (Figure 3A). Thus, Sestrin2 mutants that do not bind to WDR24 could not signal the absence of leucine to mTORC1.

To understand the molecular mechanisms underlying the insensitivity of mTORC1 towards leucine, MD simulations were conducted. The system contained Sestrin2 and WDR24 Nβ in an orthogonal water box, where mutations were performed on WDR24 (R46E-K120E-R167E), Sestrin2(L351D-D346R-D364R) and wild-type proteins, comprising three independent sets of MD runs (Figure 3B). Due to the presence of side-chain mutation, trajectories of three MD runs were firstly aligned on the backbone of WDR24 Nβ, then RMSD of three sets was calculated by backbone of Sestrin2 with reference of the backbone of Sestrin2 wild-type after equilibrium. The high RMSD value at the beginning of production run of sets indicates a shift of the binding interface influenced by triple mutants (Figure 3C). The significant interface deviation probably caused fatal torsion of the GATOR2 scaffold, forcing the interfaces of WDR24 and other parts of GATOR2 to shift away from their native state and weakening the stability of the entire GATOR2 complex. This may explain the resistance of mTORC1 to leucine deprivation caused by the Sestrin2 mutants (Figure 3A).

These biochemical results combined with the AF2 models provide interesting hypotheses that can be further tested experimentally. Notably, Sestrin2 was proposed to utilise separate domains (NTD and CTD) to exert independent functions, NTD for the regulation of reactive oxygen species and CTD for the control of mTORC1 signaling [37]. However, single site Ser190 mutant within Sestrin2 NTD was proved to considerablely decrease the leucine-sensing ability of mTORC1 [11,20], which is in agreement with the interface of Sestrin2-Seh1L in the predicted model (Supplementary Figure S4). Consistent with the predicted structural model, rational Sestrin2^339-480^-WDR24^Nβ^ interface was found and the mutations of aforementioned reisdues leads to an attenuated Sestrin2–WDR24 interaction. Further work will be necessary to structurally validate the AF2-multimer prediction and to explore the function of both NTD and CTD from Sestrin2 in the regulation of mTORC1.

### Insights into the interaction between CASTOR1 and Mios Nβ using the predicted model

Previously, we resolved the crystal structure of CASTOR1 and demonstrated that out of five GATOR2 subunits, only Mios associates directly with CASTOR1 *in vitro* [21]. Later, it was suggested that Mios Nβ alone is sufficient to interact with CASTOR1 [18].

**Figure 4:**
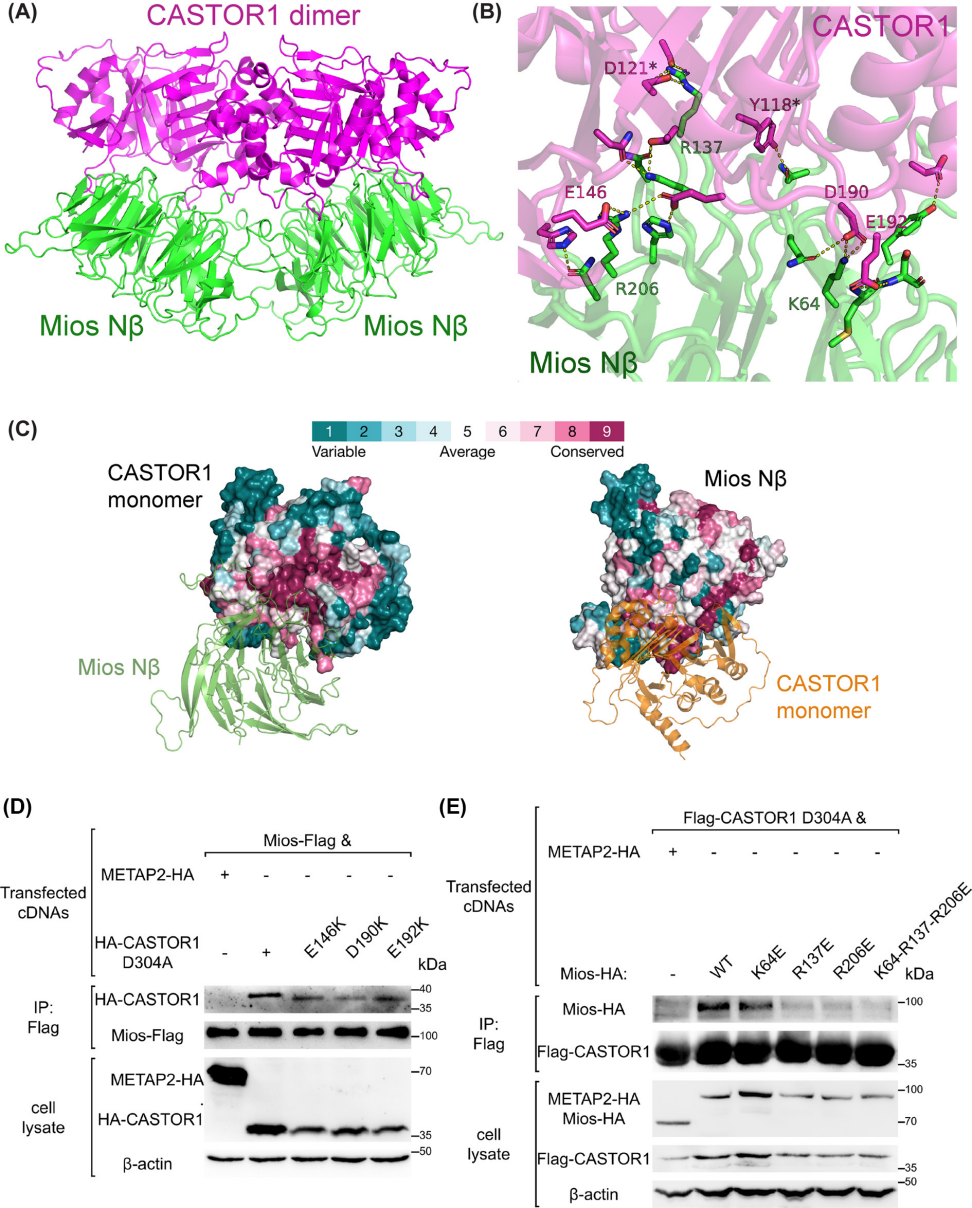
AF2 model of CASTOR1-Mios Nβ is validated by interface analyses (**A**) Cartoon representation of CASTOR1 dimer and Mios Nβ as predicted by AF2-multimer. CASTOR1 (coloured in magenta), Mios Nβ (coloured in green). (**B**) The interface of CASTOR1-Mios^Nβ^. Previously reported residues for GATOR2-binding, D121 and Y118, are indicated by asterisks. (**C**) ConSurf analyses for CASTOR1 (left) and Mios β-propellor (right) as shown by surface. (**D**) The binding of METAP2 (as a negative control), CASTOR1^D304A^, and different CASTOR1-based mutants to Mios was assessed via anti-Flag co-IPs. (**E**) The binding of METAP2, Mios and different Mios mutants to CASTOR1^D304A^ was evaluated by anti-Flag co-IPs. The results in (**D,E**) are representative of three independent experiments.

**Figure 5:**
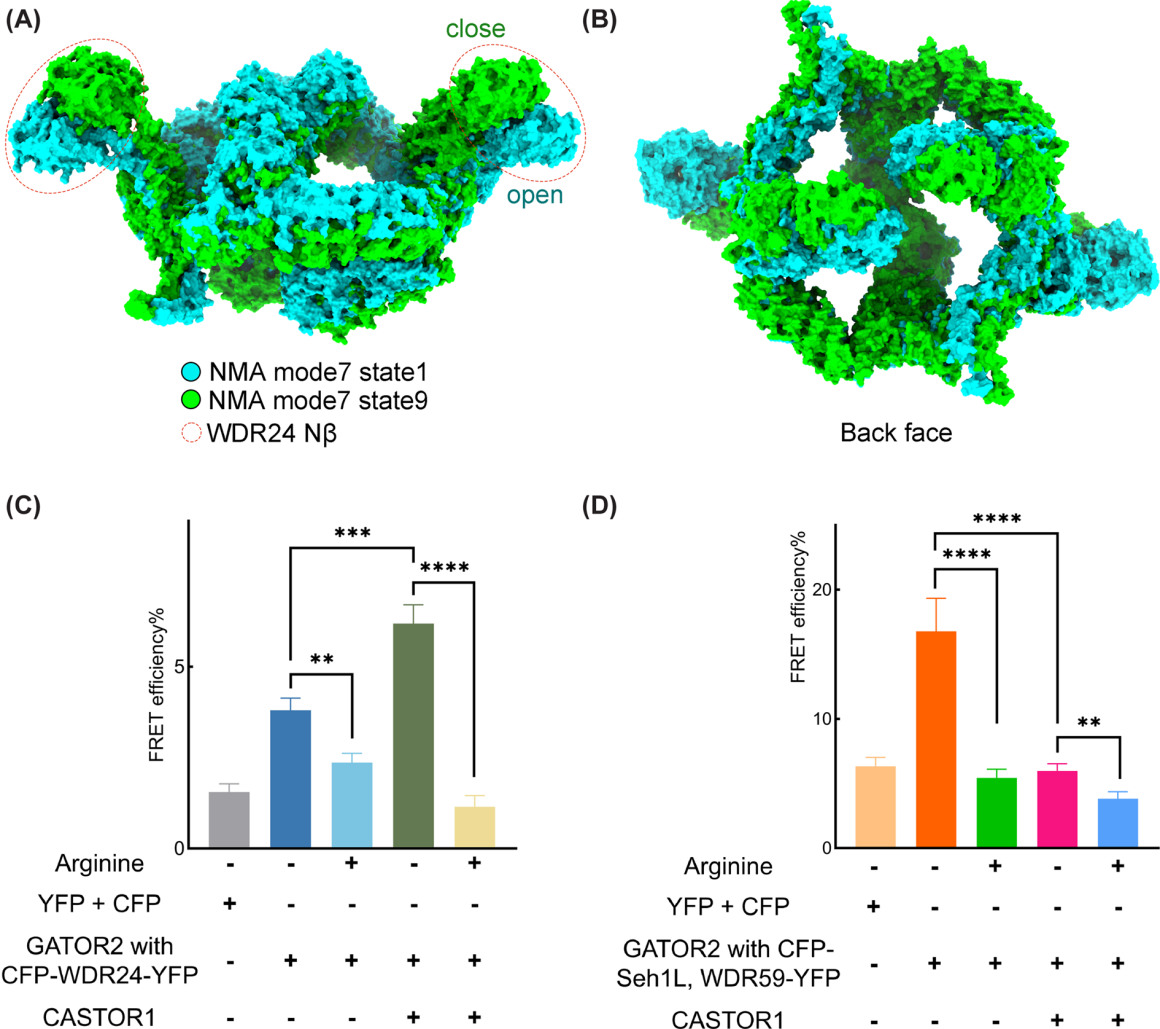
NMA and FRET analyses reveal the conformational changes in GATOR2 (**A**) The alignment of two normal mode states from the 7th normal mode was visualized by USCF ChimeraX program with the side view of GATOR2. (**B**) Aligned normal mode states showing the back face of GATOR2. (**C**) FRET efficiency detected by the expression of CFP-WDR24-YFP and other indicated proteins under arginine deprivation or re-stimulation. Two pRK7 plasmids expressing CFP and YFP, respectively, were used as a negative control. Error bars, mean ± sd, *n*≥15. *P<0.05, **P<0.01, ***P<0.001, ****P<0.0001. (**D**) FRET efficiency detected by the expression of CFP-Seh1L and WDR59-YFP with other indicated proteins. Other treatments were the same as in (C).

While the high-resolution structure of GATOR2 (PDB: 7UHY) and CASTOR1 (PDB: 5I2C) are known, it’s unclear how Mios structurally engage with CASTOR1. And it’s also unknown whether the interaction involves two Mios Nβs on the side of GATOR2 architecture to spatially accommodate CASTOR1 dimer. Given that arginine sensor CASTOR1 functions as a homodimer to inhibit GATOR2 in mammals [10,13,21], we applied the AF2-multimer algorithm to obtain a model of CASTOR1 dimer with two copies of Mios Nβ (residues 1–352), constituting four chains when submitted to AF2-multimer. Among the five models, the best-ranked model was choosen by the same three metrics as mentioned above (Figure 4A and Supplementary Figure S5A). As shown in the interface of CASTOR1-Mios complex, Tyr118 and Asp121 from CASTOR1 were predicted to interact with Mios Nβ (Figure 5B), as evidenced to be required for GATOR2-binding in previous work [13,21]. In the top-ranked model, aa residues 321-329 in the C-terminus of CASTOR1 had 55 < pLDDT < 80 (Supplementary Figure S5B). Another exposed loop (residues 210–223) showed a low pLDDT value (<60). Remaining regions of CASTOR1 were estimated to be correctly modelled. For Mios Nβ, the propeller core revealed high confidence, with pLDDT values ranging from 70.65 to 92.39, except for peripheral disordered loops (Supplementary Figure S5B). Hence, the predicted model of CASTOR1 and Mios Nβ were used in the present study for further analysis.

The ConSurf web server was employed and the results showed a highly conserved interface between Mios Nβ and CASTOR1 (Figure 4C). The AF2 prediction suggested that previously undiscovered residues contribute to stable associations between Mios Nβ and CASTOR1 (Figure 5B). These were mainly electrostatic interactions with salt bridges formed by Mios^K64^-CASTOR1^D190^, Mios^R137^-CASTOR1^D121^, Mios^R206^-CASTOR1^E146^, and Mios^K5^-CASTOR1^E192^. Multiple sequence alignments indicated that these residues are evolutionarily conserved (Supplementary Figure S5C,D). To test binding contributions, negatively charged residues were mutated into positively charged residues and vice versa. Interface ΔΔ*G* values were predicted by flex ddG and showed destabilising effects on the Mios-CASTOR1 interface (Supplementary Figure S5E). Given the weak interactions between wild-type CASTOR1 and Mios [21], the CASTOR1 D304A mutant was used to perform co-IP experiments to discharge arginine and constitutively bind with GATOR2 [13,21,38,39]. The mutants of CASTOR1 (E146K, D190K and E192K), though poorly expressed as soluble components in Expi293F cells, had significantly impaired binding affinity (Figure 5E). The Mios mutants R137E and R206E also largely inhibited complex formation, whereas K64E had a moderate deterrent effect (Figure 5F).

Together, the insights from the AF2-multimer prediction showcase a possible structural arrangement for CASTOR1 dimer and Mios Nβ, and identify previously unfound residues involved in binding by mutagenesis testing.

### Conformation changes of GATOR2 in response to arginine

GATOR2 complex forms an octagon arrangement with the outer expansion of β-propeller pairs comprising WDR24^Nβ^-Seh1L and WDR59^Nβ^-Sec13, and Mios^Nβ^-Seh1L extrudes into the centre of the octagon [18]. To understand the structural dynamics of GATOR2, its intrinsic flexibility was illustrated by normal-mode analysis (NMA) using WEBnm online software [40,41]. Based on the trajectories of the lowest-frequency mode, the overall conformational motion of GATOR2 mimics the movement of a hairpin from ‘open’ to ‘closed’ state (Figure 5A,B; Supplementary Movie S1 and S2). Atomic displacements represent the dominant aspect of dynamics based on normal-mode theory [42]. Remarkably, WDR24 Nβ contributed to the highest displacement values (Figure 5A and Supplementary Figure S6), and two Mios Nβs on the back face and Seh1L^WDR24^ (one β-blade donated by WDR24) also showed remarkable shifts (Figure 5B and Supplementary Figure S6). NMA provides structural insights into the potential movement of the outer β-propellers within GATOR2, which leads to a presumption of a conformational change in GATOR2 triggered by amino acid sensors.

To corroborate this hypothesis experimentally, we attempted to detect the FRET by constructing fluorophore-conjugated proteins with cyan fluorescent protein (CFP) fused to the N-terminal end of Seh1L (CFP-Seh1L) and yellow fluorescent protein (YFP) attached to the C-terminal end of WDR59 (WDR59-YFP), respectively. To eliminate selection bias, YFP and CFP were fused to the N-terminal end and C-terminal end of WDR24 (YFP-WDR24-CFP). The introduction of YFP and CFP was verified to have little effect on the formation of GATOR2 complex (Supplementary Figure S7A) and visualized using microscope to ensure effective expression (Supplementary Figure S7B). There was a tendency, as tested via YFP/CFP-labelled constructs, for the FRET efficiency to be significantly lower when arginine was abundant than when it was depleted, even when CASTOR1 was not over-expressed in cells (Figure 5C,D). This may be because CASTOR1 present in the background of HEK-293T cells binds to GATOR2 during arginine starvation. In an arginine-free state, the addition of CASTOR1 caused a FRET change (Figure 5C,D). This is thought to explain the effect of CASTOR1-binding on GATOR2 conformation. It is worth noting that in the group using CFP-Seh1L and WDR59-YFP (Figure 5D), in the absence of arginine and external CASTOR1, the FRET efficiency in the second column is much higher than that in the group using CFP-WDR24-YFP (Figure 5C). This is most likely due to the close proximity of Seh1L and WDR59 in GATOR2 (near 58 Å) compared with the distance of WDR24 terminals (approximately 78 Å).

These same constructs were used to detect FRET responses at different leucine levels. After co-transfection of expression plasmids of GATOR2 with Sestrin2, variations in leucine level caused changes in FRET efficiency (Supplementary Figure S7C). Although the significance level tended to be lower than that for CASTOR1, the decreasing trend of the FRET efficiency upon leucine treatment was consistent with that of the co-transfection of CASTOR1 and GATOR2 in response to arginine.

### Mios Nβ is essential to trigger the conformational change in GATOR2

**Figure 6:**
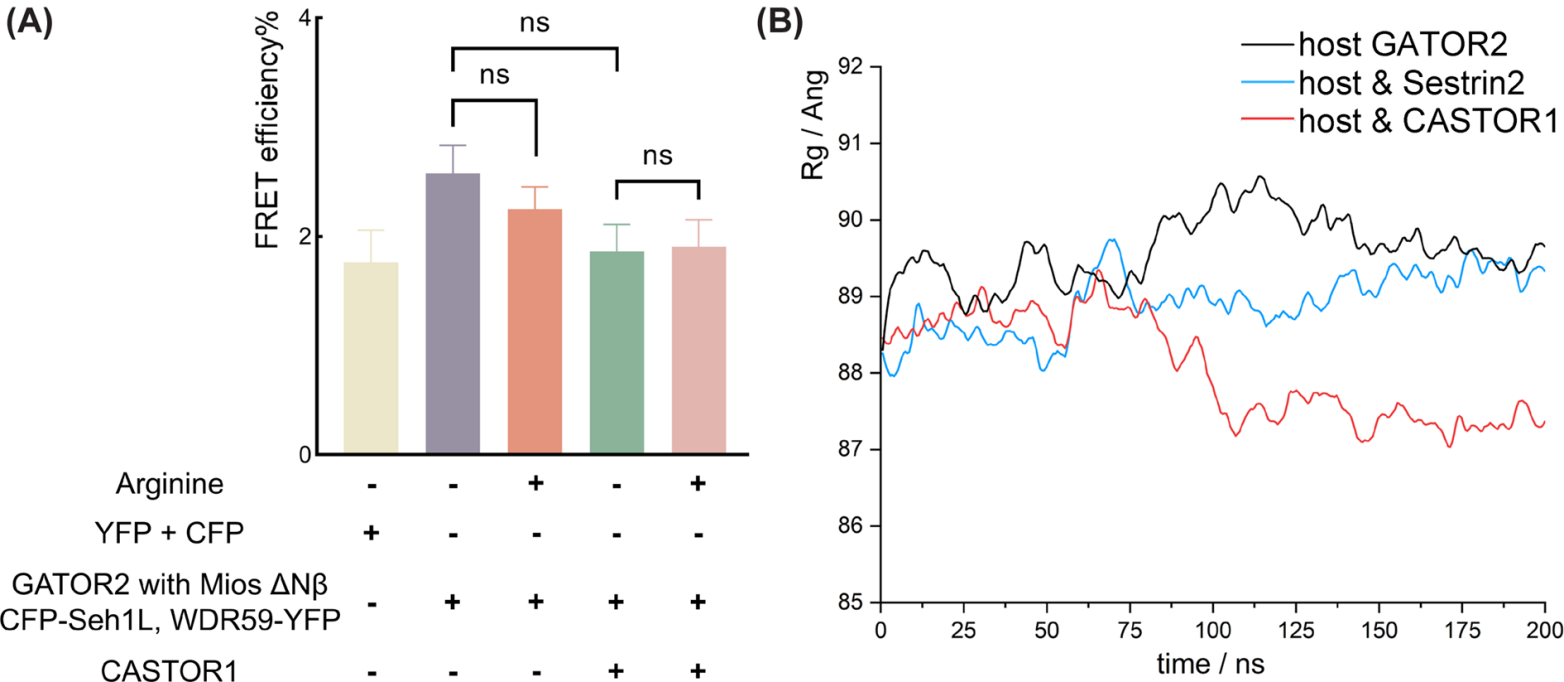
Deletion of Mios Nβ diminishes the conformational change in GATOR2 (**A**) Introducing the deletion of Mios β-propeller in the GATOR2-expression constructs. Other experimental treatments to detect FRET efficiency were kept the same as in Figure 5C,D. (**B**) *R*_g_ of GATOR2 generated from three independent MD runs: GATOR2 only (coloured in black), Sestrin2-bound GATOR2 (coloured in cyan). CASTOR1-bound GATOR2 (coloured in red).

As Mios Nβ interact directly with CASTOR1 [18], we hypothesised that CASTOR1 requires the β-propeller of Mios to trigger the conformational change in GATOR2. To test the prediction, we next designed a Mios Nβ deletion mutant and a WDR24 Nβ deletion mutant, respectively. Although the integrity of GATOR2 complex was unaffected by the deletion of Mios Nβ (Supplementary Figure S8A), truncation of WDR24 Nβ caused a weakened association of GATOR2 subunits compared with full-length WDR24 (Supplementary Figure S8A). Thus, we compared the efficacy of Mios Nβ deletion with that of full-length Mios. Consistent with the prediction, the deletion of Mios Nβ completely undermined the FRET change in response to arginine in the presence or absence of overexpressed CASTOR1 (Figure 6A). Therefore, Mios Nβ is indispensable to the conformational transition of GATOR2.

After aligning the predicted models, Sestrin2-WDR24-Seh1L and CASTOR1-Mios Nβ, to the corresponding areas of GATOR2 (Supplementary Figure S8B,C), two sets of MD simulations were performed to probe the molecular effect of the amino acid sensors on GATOR2. GATOR2 coupled with CASTOR1 or Sestrin2 was subjected to conventional MD simulations. As the complex is too large for conventional MD simulation to generate notable conformational changes, Gaussian-accelerated MD (GaMD) simulations were applied to perform enhanced and unconstrained conformational sampling. While GaMD is unable to perform statistical analysis of time-dependent properties, it samples free energy landscape and related nature thoroughly compare to conventional MD. The difference in the radius of gyration (*R*_g_) showed that a significantly reduced Rg in GATOR2 in complex with CASTOR1 compared with that in the non-binding state (Figure 6B). However, in complex with Sestrin2, the *R*_g_ of GATOR2 showed no distinct change compared with the unbound state. MD simulations indicated increased flexibility of the unbound GATOR2 possibly corresponding to the open conformation (Figure 5A), and CASTOR1-bound GATOR2 had a more compact conformation than Sestrin2-binding GATOR2 (Figure 6B). These findings strongly support the results of FRET analyses (Figure 5C,D and Supplementary Figure S7C), in which CASTOR1 led to a more significant conformational change in GATOR2.

Together, FRET experiments revealed that the potential structural transition of GATOR2 can be triggered by CASTOR1 and Sestrin2 and the interaction of CASTOR1-Mios Nβ is essential for the conformational change of GATOR2 in response to arginine signal (Supplementary Figure S8C).

## Discussion

GATOR2 complex, acting as a nutrient-sensing hub in mTORC1 pathway, is directly regulated by amino-acid-sensing proteins including leucine sensor Sestrin2 and arginine sensor CASTOR1. Focusing on the interactions between GATOR2 and amino-acid-sensing proteins, AF2-multimer was employed to generate predicted models of Sestrin2-WDR24-Seh1L (Figure 1B) and CASTOR1-Mios Nβ complexes (Figure 4A). Based on the determined crystal and cryo-EM structures available in the PDB, individual domains in these models showed high pLDDT scores (Figure 1C and Supplementary S5B). To assess the binding interfaces, mutational studies were conducted to validate the interactions using the Rosetta saturation mutation methodology (Supplementary Figures S2C and S5E) and biochemical experiments including IPs (Figure 3A) and co-IPs (Figures 2C,D and 4D,E). Through normal-mode analysis (Figure 5A,B) and molecular dynamics simulations (Figure 6B), possible motions inherent in the GATOR2 structure were identified, which was further supported by FRET experiments carried out in cells using an established CFP-YFP methodology (Figure 5C,D).

Based on previous studies, the β-propeller domains of WDR59, Mios, WDR24, Seh1L and Sec13 are essential for amino-acid-induced mTORC1 activation [18,20]. WDR24 forms a complex with Seh1L via donating an extra β-blade to complete the open β-propeller of Seh1L [18]. Structurally, this complex accommodates Sestrin2 with good shape complementarity, which is a novel discovery in the present study. Under physiological condition, leucine stimulation was suggested to turn Sestrin2-bound WDR24^RING^ into UBE2D3-binding with subsequent ubiquitination of NPRL2, depicting a role of GATOR2 in GATOR1 inactivation [20]. Although the RING domain of WDR24 in the top-ranked model showed no direct contact with Sestrin2 (Figure 1C), the top five predictions differ in the relative orientation of the RING and α-solenoid domain of WDR24 (Supplementary Figure S1A). This relates to a possible flaw in predicting the spatial arrangement for multidomain proteins, especially when separated by unstructured regions [43]. Thus, it is possible that the loop in between is pliable for WDR24 RING domain to engage with Sestrin2 and regulate GATOR1 *in vivo*. Additionally, Sestrin2 Ser190, locating in the interface of Sestrin2^NTD^-Seh1L (Supplementary Figure S4), was proved to be essential for WDR24-binding, and NPRL2 was constitutively ubiquitinated when introducing Sestrin2^S190^ mutant [20]. Combined with the contributions of Sestrin2 (L351, D346 and D364) to WDR24-binding (Figure 2C), it indicates that the three interfaces, including WDR24^Nβ^-Sestrin2, WDR24^RING^-Sestrin2, and Seh1L-Sestrin2, are equally important to the ineraction of Sestrin2–GATOR2 and the downstream regulation towards NPRL2.

Following our previous *in vitro* pull-down experiments using bacterially purified proteins [21], the predicted model of CASTOR1 and Mios Nβ complex showed that CASTOR1 dimer engages with two copies of Mios Nβ (Figure 4A). It proposes an intriguing assembly for CASTOR1-GATOR2 complex, where CASTOR1 dimer meets with the two Mios propellers on the one side of the GATOR2 structure. Due to the four copies of Mios within GATOR2, whether the interaction of CASTOR1-Mios happens in the back face, front face, or both sides of GATOR2 architecture is unknown (Figure 5B and Supplementary Figure S8B); however, the physical distance of adjoining Mios Nβs in the front face seems to be a suitable platform for CASTOR1 dimer (Supplementary Figure S8B), which requires further investigation into the topological structures of CASTOR1 and GATOR2 using techniques such as small-angle X-ray scattering.

Conformational changes of GATOR2, as implied in one mechanistic study [20], may be caused by the dissociation of Sestrin2 and further play a role in the regulation of GATOR1. However, one major drawback of AF2 prediction is that the it only generates a static structure without information about the possible motions, which leaves room for the exploration of the protein structure-function relationship. In this study, movies from NMA revealed a high degree of motion from the central Mios β-propeller pairs and the peripheral propellers of WDR24 (Supplementary Movies S1 and S2). In addition, we detected prominent conformational changes of GATOR2 in cells in response to amino acid stimulation and the interaction with sensors (Figure 5C,D and Supplementary Figure S7C), providing convicing evidence for the link between the native dynamics of GATOR2 and its physiological function. Given that GATOR2 negatively regulates GATOR1 in response to amino acids [12,44], the dynamics of GATOR2 may also play a role in the regulation of GATOR1. Although the cryo-EM structure of the yeast GATOR complex (SEAC) has been resolved, critical residues for the connection between GATOR1 and GATOR2 are not conserved in mammals [45]. Without structural information, it remains a mystery how GATOR2 interacts with GATOR1 via WDR59 Nβ [18]; similarly, whether the conformational changes of GATOR2 relate to its manipulation to the ubiquitination of NPRL2 upon amino acid stimulation requires solid GATOR2-based structural and functional research.

## Methods

### Antibodies and reagents

HPR-conjugated anti-rabbit and mouse secondary antibody (catalog no. M21003) was from Abmart and used at 1:5000 dilution. Antibodies against Flag-tag (catalog no. M20008), HA-tag (catalog no. M20003), and β-actin (catalog no. T40104) were from Abmart and used at 1:2000-1:5000 dilution. Antibodies to mTOR (catalog no. 2972S), phospho-T389 S6K1 (catalog no. 9205S) were purchased from Cell Signalling Technology and used at 1:1000 dilution. Antibody against GFP (catalog no. 50430-2-AP) was from Proteintech and used at 1:1000 dilution. All antibodies were diluted in 1% BSA dissolved in TBST (25 mM Tris-HCl, pH 8.0, 150 mM NaCl, 0.05% Tween-20).

Anti-FLAG M2 affinity gel (catalog no. A2220) was from Sigma Aldrich. ExpiFectamine293 Transfection Kit (catalog no. A14525) and trypsin-EDTA (catalog no. 25300062) were purchased from Thermo Fisher Scientific. Dulbecco’s modified Eagle’s medium (DMEM, catalog no. 10-013-CV) was from Corning. Insect cell SIM SF (catalog no. MSF1) and SIM HF (catalog no. MHF1) expression medium were from Sino Biological. Foetal bovine serum (FBS, catalog no. SFBS-X) was from BOVOGEN. cOmplete Mini protease inhibitor cocktail (catalog no. 11836170001) was from Roche. Pre-stained protein ladder (catalog no. P8032M) was from UElandy. Leucine- or arginine-deficient RPMI medium was made in our institute following product information online: https://www.sigmaaldrich.com/deepweb/assets/sigmaaldrich/product/documents/985/367/r7130dat.pdf

### Plasmids constructs

The pRK5 plasmids expressing five subunits of GATOR2, CASTOR1 and YFP were from previous study [21,46]. The cDNA encoding human Sestrin2 was chemically synthesized (Union-Biotech, Shanghai). The cDNA encoding METAP2 was obtained from cDNA libraries. For pRK7 constructs as used in the purification of WDR24, Seh1L and Sestrin2 (Figure 1A), PCR products and plasmids were cut using the same enzymes and joined via T4 DNA ligase (NEB). For site-directed mutagenesis, all the primers are listed (Supplementary Table S1), and a one-step cloning kit (Novaprotein, catalog no. NR005-01B) was used to insert PCR products into pRK5 that had been digested with *Hind*III and *BamH*I. Double or triple mutants were constructed using previously prepared single-site mutants as templates. With the cDNA template of YFP, the construct for CFP was generated using site-directed mutagenesis. The PCR products of CFP/YFP and Seh1L/WDR59/WDR24 were cloned into pRK7 using one-step cloning kit (Novaprotein). All constructs were verified by sequencing.

### Protein purification

The pRK7 plasmids expressing N-terminal MBP-WDR24, Seh1L-His, and Sestrin2-HA were co-transfected into a suspension of Expi293F cells when the cells reached a density of 3 × 10^6^ cells/ml and were later purified using a dextrin-Sepharose high performance affinity column (GE Healthcare, catalog no. 28-9355-98). The bound proteins were eluted and checked on Coomassie-stained SDS-PAGE gels. Yields of the proteins were subjected to Superose6 Increase 10/300 GL column chromatography (Cytiva, catalog no. 29091596). Peak fractions were analysed by western blotting using the corresponding tag antibodies.

### Cell lysis, immunoprecipitation (IP) and co-IP

The cells were rinsed with ice-cold PBS and lysed with lysis buffer (25 mM Tris-HCl, pH 8.0, 150 mM NaCl, 1% NP40, 1 mM MgSO_4_ and one tablet of protease inhibitor per 50 ml buffer). The cell lysates were separated by centrifugation at 14,000 rpm at 4°C for 30 min. The FLAG-M2 affinity beads were added to the supernatant and incubated by rotating for 3 h at 4°C. The beads were washed four times with TBS (25 mM Tris-HCl, pH 8.0, 150 mM NaCl). Bound proteins were denatured with SDS sample loading buffer and boiling for 5 min and then analysed by immunoblotting.

For IP experiments, about 0.8 million human embryonic kidney 293T (HEK-293T) cells were seeded in a 6-cm cell culture dish. After 24 h, HEK-293T cells were transfected using the polythylenimine method with pRK7 Flag-S6K1, pRK7 HA-METAP2 (as a negative control), pRK7 HA-CASTOR1 WT, or single-site mutants, and pRK7 HA-Sestrin2 WT or mutant cells were collected 36–48 h post-transfection and lysed as described above.

For the co-IP experiments, 25 ml of Expi293F cells (density: 3 × 10^6^ cells/ml) were co-transfected with the following paired pRK7-based cDNA expression plasmids: Flag-METAP2, Flag-WDR24 WT or mutants, HA-Sestrin2 Y375F, Mios-HA, Flag-CASTOR1, Mios-Flag, and HA-CASTOR1 D304A. The cells were harvested 48 h post-transfection and lysed as described above.

For experiments that required arginine/leucine starvation and re-stimulation, HEK-293T cells were rinsed with arginine/leucine-free RPMI, incubated with the same medium for 50 min and then re-stimulated with 500 μM arginine/leucine for 10 min.

### Western blotting analysis

For the co-IP experiments, the protein concentration in cell lysate was determined using the BCA assay (Solarbio Life Sciences, catalog no. PC0020). Fractions separated by 10% SDS-PAGE were electrotransferred on to 0.22 μm PVDF membrane (Sigma Aldrich, catalog no. ISEQ00010) at 100 V for 90 min using a tank transfer unit (Bio-Rad) and a transfer buffer comprising 50 mM Tris-HCl, 40 mM glycine and 20% methanol. The membranes were blocked with 5% (w/v) skim milk in 25 mM Tris-HCl, 150 mM sodium chloride and 0.1% Tween-20, pH 7.4. Blots were probed with antibodies of interest with the recommended dilution of product information in blocking solution overnight at 4°C, followed by incubating with HRP-conjugated goat anti-rabbit/mouse secondary antibody in blocking solution at 4°C for 3 h. The signal was detected using ECL western blotting substrate (Abmart, catalog no. A10016S).

### FRET analysis

Each FRET experiment in the present study was performed on the same batch of HEK-293 cells cultured on coverslips and transfected with pRK7-based cDNA expression plasmids, including different combinations of HA-tagged CASTOR1, HA-tagged Sestrin2 and GATOR2 constructs (with CFP-WDR24-YFP or CFP-Seh1L/WDR59-YFP). At 48 h after transfection, cells were subjected to arginine or leucine deprivation, re-stimulated as previously described, immediately rinsed twice with PBS, and fixed in 4% formaldehyde for 20 min at room temperature. Fluorescence signals of the CFP (donor dye) and YFP (acceptor dye) molecules were acquired using a Leica TCS SP8 Confocal microscope. The CFP signal was obtained once before photobleaching (BP) and once after photobleaching (AB) the YFP at the top of each cell, with the bottom half of each cell used as the internal control. Each dataset is based on >15 cells. In each cell, three-four regions of interest in the photobleached area were selected for analysis. FRET efficiencies were determined automatically using the Leica FRET AB software using the following formula:

$$\text{FRET}\%=\left(\frac{\left(\text{CFPAP}-\text{CFPBP}\right)}{\text{CFPAP}}\right)\times 100$$

Normality tests were performed for each group, and statistical significance was determined using an unpaired *t*-test in GraphPad Prism (version 8.0.2). Throughout, the significance was reported at *P*≤0.05, and the data were presented as mean ± standard deviation.

### Structure prediction using AlphaFold2-Multimer

The structural models of the Mios^Nβ^-CASTOR1 and Sestrin2-WDR24-Seh1L complexes were predicted using the AF2-multimer [30]. The predictions were performed on the super performance platform of Shanghai Jiaotong University (Shanghai, China) and AlphaFold2 version2.3.1 was employed with a cut-off date of 14 May 2023, and details of the parameters are given in the supplementary information. The confidence of AF2 models were evaluated by pLDDT and predicted aligned error (PAE), which were visualized using UCSF ChimeraX program [47]. Alignments of AF2 predictions on released structures were analysed using the align tool of PyMOL version 2.5.5 (https://pymol.org/2/).

### Normal-mode analysis (NMA)

The structural coordinates of GATOR2 (PDB ID: 7UHY) were submitted for NMA using WEBnm@ v2.0 [40,41]. In the output, the 7th normal mode in this list, which is the 1st vibrational mode (the first six normal modes are zero-frequency modes corresponding to the global rotation and translation of the system), is the slowest in terms of vibrational frequency and is thus regarded as the most important vibrational mode. The 7th normal mode was selected for further analysis.

### Cell culture

Expi293F and HEK-293T cells were maintained at 37°C with 5% CO_2_. Expi293F cells were cultured in Expi293 Expression medium (catalog no. A1435101) at 125 rpm; HEK-293T cells were cultured in DMEM supplemented with 10% FBS and penicillin-streptomycin (Thermo Fisher Scientific, catalog no. 15140122).

### Interface ΔΔG values using flex ddG

The ddG mutation of the saturation mutation was calculated via flex ddG [48], in which a single-site mutation was performed, followed by local configuration space sampling of the nearby side chain and main chain. The ΔΔ Gibbs free energy (dd*G*) of interface binding was calculated to demonstrate the contribution of the mutation. The energy terms were sampled and scored using Rosetta Scripts. Mutation sites were picked among all nearby residues within 5 Å from the other surface of protein–protein interface. Flex ddG results were analysed using analyze_flex_ddG.py from the flex_ddG GitHub page and visualized using the Python package seaborn.

### Molecular dynamics simulation

The missing residues in the GATOR2 structure (PDB ID: 7UHY) were supplemented in PyMOL via the Builder panel, based on the protein sequence of each subunit, and automatically modelled using ModLoop [49]. The input structures were prepared using pdb4amber and tleap, which are the shell scripts supplied by AMBER. Conventional MD and Gaussian-accelerated MD simulations were performed using the AMBER software [50]. The TIP3P and ff14SB force fields were applied to water and proteins [51]. Each system underwent a two-step EM for the solvent and entire system sequentially, followed by heating from 0 to 300 K for 50 ps. Each system was equilibrated under an isothermal-isobaric ensemble (300 K and bar) for 50 ns for density convergence to obtain the initial conformation for the final production run. The production run lasted for at least for 100 ns to collect data such as the root mean square deviation (rmsd), *R*_g_, and hydrogen binding. The SHAKE algorithm was applied after EM for all bonds formed with hydrogen atoms. The Particle Mesh Ewald method was used to describe the long-range electrostatic interactions [52]. Cpptraj and MDAnalysis were used to analyse the MD trajectories.

## Supplementary Material

Supplementary Figures S1-S8 and Table S1

Supplementary Movies S1 and S2

## Data Availability

To support the findings in this study, the data are available within the article and its Supplementary Materials.

## Abbreviations

AF2-multimer: AlphaFold2-Multimer
CFP: cyan fluorescent protein
co-IP: co-immunoprecipitation
cryo-EM: cryo-electron microscopy
FRET: fluorescence resonance energy transfer
GAP: GTPase-activating protein
GEF: guanine-nucleotide exchange factor
GTPase: guanosine triphosphatases
HEK-293T: human embryonic kidney 293T
IP: immunoprecipitation
MD: molecular dynamics
mTORC1: mechanistic target of rapamycin complex 1
NMA: normal mode analysis
Nβ: N-terminal β-propeller
*R*_g_: radius of gyration
rmsd: root mean square deviation
YFP: yellow fluorescent protein
