# Supplementary material for: New insights into GATOR2-dependent interactions and its conformational changes in amino acid sensing
Source: Biosci Rep. 2024 Mar 13;44(3):BSR20240038. doi: 10.1042/BSR20240038 (PMC10938194; doi:10.1042/BSR20240038)
Supplement: Supplementary Figures S1-S8 and Table S1 [file BSR-2024-0038_supp.pdf]

Supporting Information for

New insights into GATOR2-dependent interactions and its conformational changes in amino acid sensing

Can Yang[12], Xuan Sun[12], Geng Wu[1]*

[1]State Key Laboratory of Microbial Metabolism, School of Life Sciences & Biotechnology, the Joint International Research Laboratory of Metabolic & Developmental Sciences MOE, Shanghai Jiao Tong University, Shanghai, China

[2]Co-authors: Can Yang, Xuan Sun

*Corresponding author: Geng Wu

Email: geng.wu@sjtu.edu.cn

TABLE OF CONTENTS

The command to run AF2-multimer with all parameters provided.

Supplementary Table S1.

Supplementary Figures S1-S8.

Movie S1 and S2.

The orginal scans of the Western blots.

The command to run AF2-multimer with all parameters provided:

```
module purge
module use /dssg/share/imgs/ai/
module load alphafold/2.1.1
singularity run --nv /dssg/share/imgs/ai/alphafold/2.1.1.sif python
/app/alphafold/run_alphafold.py \
--data_dir=/dssg/share/data/alphafold \
--bfd_database_path=/dssg/share/data/alphafold/bfd/bfd_metaclust_clu_complete_id3
0_c90_final_seq.sorted_opt \
--uniclust30_database_path=/dssg/share/data/alphafold/uniclust30/uniclust30_2018_0
8/uniclust30_2018_08 \
--uniref90_database_path=/dssg/share/data/alphafold/uniref90/uniref90.fasta \
--mgnify_database_path=/dssg/share/data/alphafold/mgnify/mgy_clusters.fa \
--template_mmcif_dir=/dssg/share/data/alphafold/pdb_mmcif/mmcif_files \
--obsolete_pdbs_path=/dssg/share/data/alphafold/pdb_mmcif/obsolete.dat \
--fasta_paths=my_fasta.fasta \
--max_template_date=2023-05-14 \
--model_preset=multimer \
--output_dir=output \
--pdb_seqres_database_path=/dssg/share/data/alphafold/pdb_seqres/pdb_seqres.txt \
--uniprot_database_path=/dssg/share/data/alphafold/uniprot/uniprot.fasta \
```

Table S1. Primers used in site-direct mutagenesis.

Primer name	sequences
5'-S2-Hind3-F	CGATGTTCCAGATTACGCTAAGCTTATCGTGGCGGACTCCGAGT
3'-S2-BamH1-R	CACCACACTGGACTAGTGGATCCTTAGGTCATG
S2-D341-346R-F	gcgcTATACCTGGGAACGCCATGGCTACTCGCTGATCCAG
S2-D341-346R-R	GCGTTCCCAGGTATAgcgCTGGGCCCGGAAGGTAGGGG
S2-D364R-F	agctgctgcgcGAGAAGTTCCAGGCAGCCTATAGCCT
S2-D364R-R	cttctcgcgCAGCAGCTGCCCACCCTCAG
S2-L351D-F	catggctacTCGgatATCCAGCGGCTTTACCCTGAGGGTG
S2-L351D-R	CGCTGGATatcCGAGTAGCCATGGTCTTCCCAGGT
5'-24-H3-F	CAAGGATGACGACGATAAGAAGCTTatggagaagatgtcccgtgtgac
3’-24-B1-R	ACCACACTGGACTAGTGGATCCtcaggagtactcgcagaggtggcc
24R46E-f	cgtggcaggcGAAagcatcttcaagatctatgccatcgagg
24R46E-r	agatgctTTCgcctgccacgaccacctgggc
24K120E-f	acagaacacGAAcgcacggtaaacaaagtctgcttcc
24K120E-r	accgtgcgTTCgtgttctgtgaacagctggtcctgc
24R167E-f	agagcgtgGAAgacgtgcagttcagtatccgggact
24R167E-r	cacgtcTTCcacgctctccgactggcccg
5’-C1-Hind3	CGATGTTCCAGATTACGCTAAGCTTGAACTGCATATCCTGGAACACCGC
3’-C1-BamH1	CACCACACTGGACTAGTGGATCCTTAGCTTGCTAAACCTTCCTGACGACG
C1-E146R-F	CATTTATCGCcgcGTTGGCGGCGAACCGGTTC
C1-E146R-R	GCCAACgcgGCGATAAATGTCGAATTCCTGCGCCA
C1-D190K-F	ACCCTGaaaCCGGAAACCTTACCGGCAATTGC
C1-D190K-R	AGGTTTCCGGtttCAGGGTTAAGACGCAAAAGCGGTTC
C1-E192K-F	TGGACCCGAAAACCTTACCGGCAATTGCAACCAC
C1-E192K-R	CGGTAAGGTTTTCGGGTCCAGGGTTAAGACGCAAAAG
5’-Mios-Sal1	CGGCCACCATGGCGGTCGACAGCGGTACCAAACCTGATATTTTATGGGC
3’-Mios-B1	GCTAAAGGCTTGGAACTGGGATC
Mios-K64E-F	ACCCTATATGGAATGTGTTGCCTGGTATCTTAATTATGA

Mios-K64E-R	TCCT AGGCAACACATTCCATATAGGGTGTATCTGAATTTATTG ACAGTAATG
R137E-F	agataagcacGAAgctgacttttcagtgctaatatgggat
R137E-R	gaaaagtcagcTTCgtgcttatctaaaccagcagctagcc
R206E-F	ggtatgcatGAAaacctagctatatttgatcttcggaatacaagc
R206E-R	agctaggttTTCatgcataccagcaaggagaagtttctg

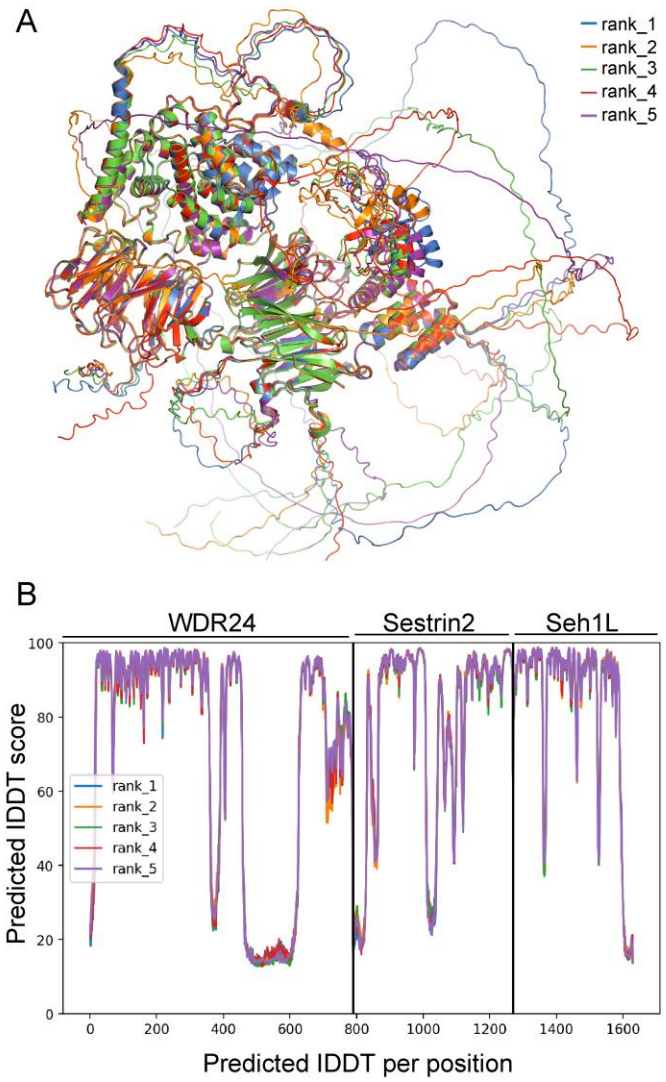

Figure S1. An overview of the Sestrin2-WDR24-Seh1L complex models. (**A**) The superimposition of the five models. (**B**) A diagram of the predicted IDDT scores. Above the diagram, the corresponding WDR24, Sestrin2 and Seh1L positions are indicated.

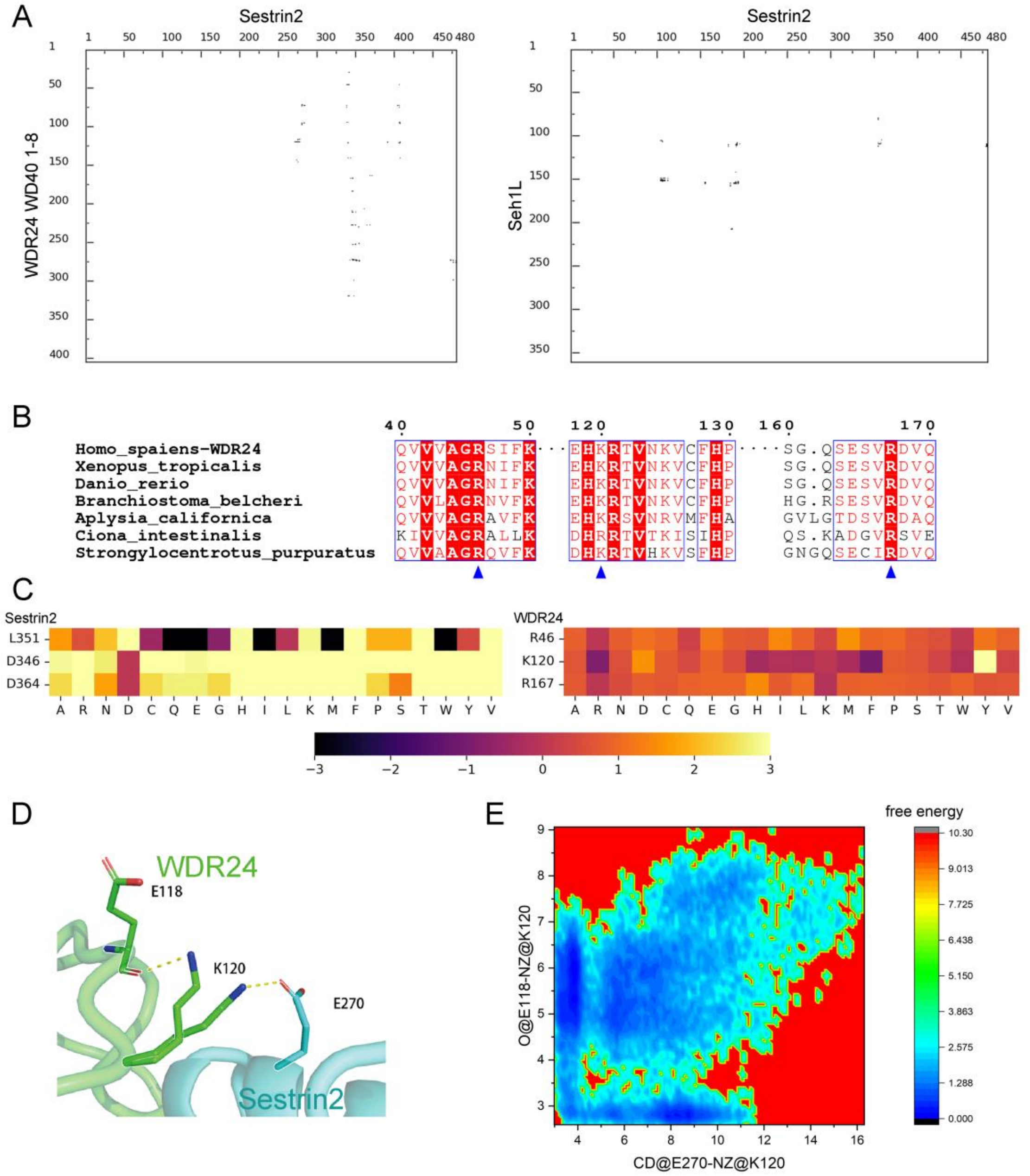

Figure S2. Analysis of the interface between Sestrin2 and WDR24 Nβ. (**A**) Atom-atom interactions within 5 Å between WDR24 1-400 and Sestrin2, or Seh1L and Sestrin2. Any interactions closer than 5 Å are presented as black dots. Online COCOMAPS tool was used to generate the contacts. (**B**) Sequence alignments of WDR24 (residues 40 to 170) from cross-kingdom species. (**C**) Flex ddG computations of saturation mutation on Sestrin2 (L351, D346 and D364) and WDR24 (R46, K120 and R167). (**D**) Two alternative binding conformations of WDR24^{K120}. Hydrogen bonds are indicated as yellow dotted lines. (**E**) Free energy landscape of

conformational distribution of K120 as shown by its distance to two different binding residues, $WDR24^{E118}$ and $Sestrin2^{E270}$.

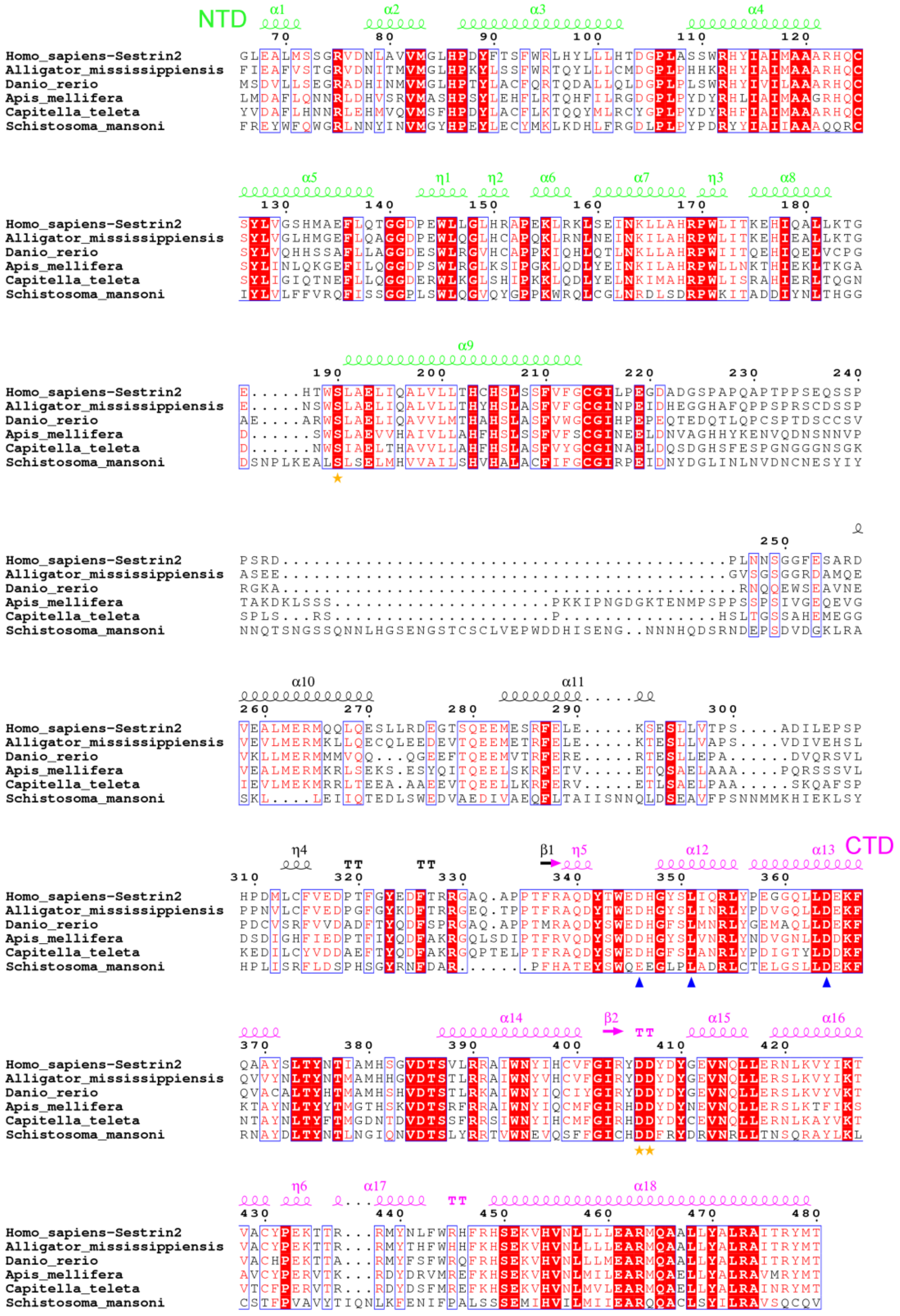

Figure S3. Sequence alignment of Sestrin2 with different homologous species. ESPript 3.0 was used to generate the alignment. The secondary structure elements of human Sestrin2 are placed on the top of the alignment where the NTD of Sestrin2 is coloured in green and the CTD is coloured in magenta. The residues selected in this

study are indicated with blue triangles. Preciously reported GATOR2-binding residues are indicated with orange stars.

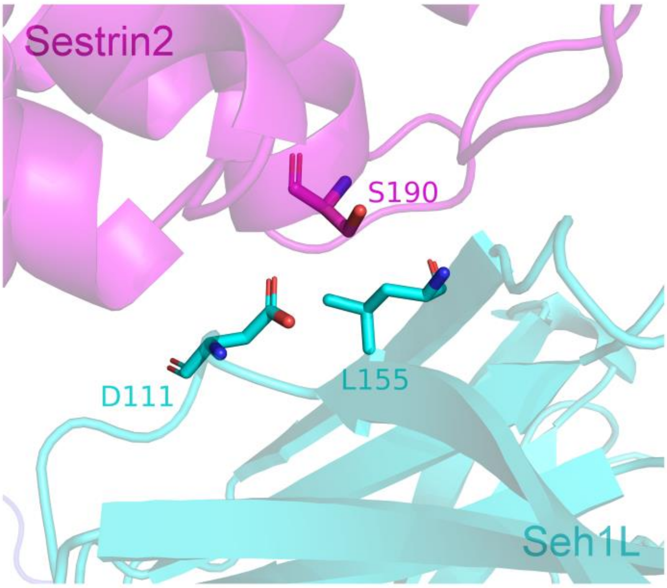

Figure S4. The binding interface between Sestrin2 and Seh1L.

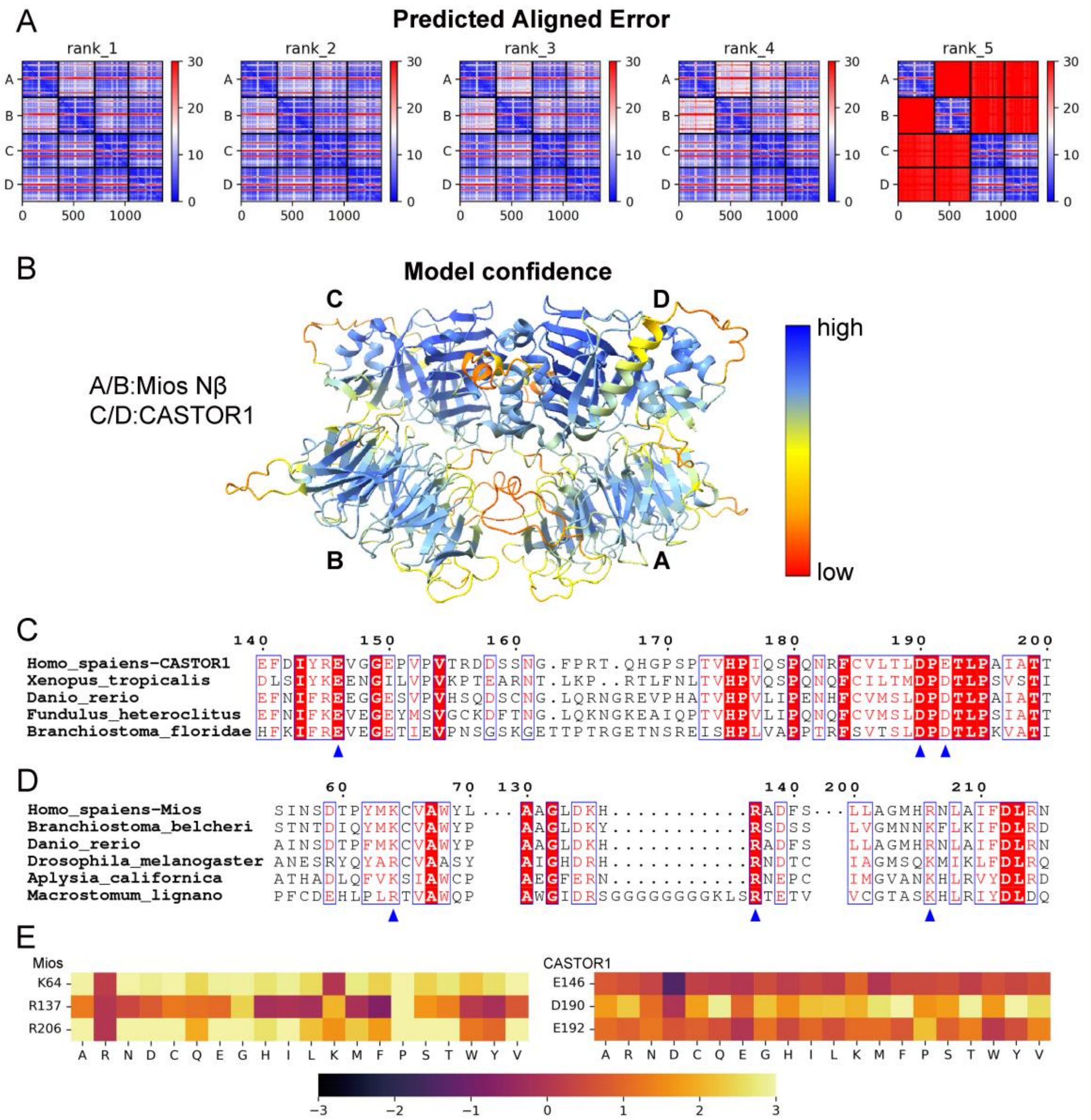

Figure S5. Assessment of the predicted model of Mios$^{N\beta}$-CASTOR1. (**A**) An overview of the Predicted Aligned Error plot from the five models of Mios$^{N\beta}$-CASTOR1 complex. The corresponding chains are indicated. Within the plot, the black lines delineate the area of individual protein of the complex. (**B**) Cartoon representation of the top-ranked model colour-coded by the pLDDT values. (**C**) Sequence alignment for CASTOR1 (residues 140 to 200). (**D**) Sequence alignment for Mios. The conserved residues marked blue triangles in (C, D) were selected for co-IP experiments. (**E**) Flex ddG computations of saturation mutations on Mios (K64, R137 and R206) and CASTOR1 (E146, D190 and E192).

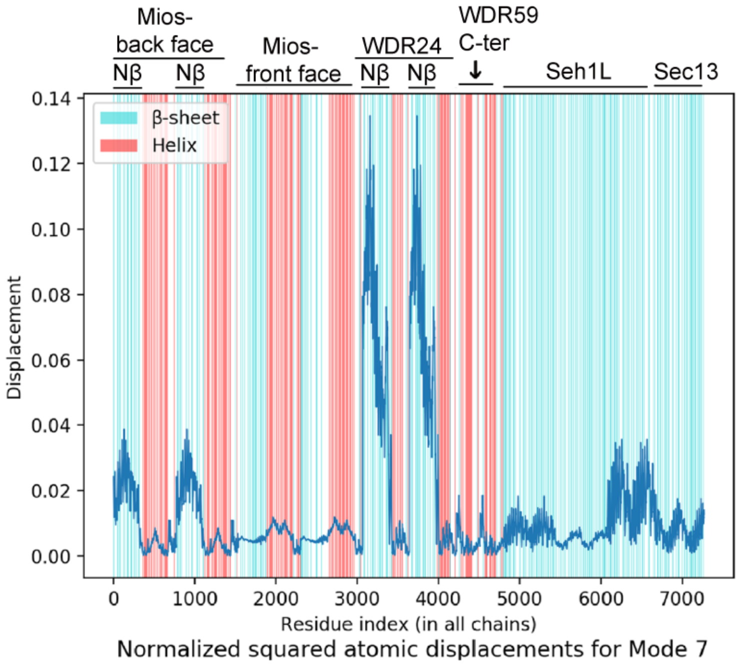

Figure S6. The 1st vibrational mode of GATOR2 NMA.

Displacement of each Cα atom in the 7th normal mode, and the corresponding subunits are indicated above. Two β-sheets of WDR24 were shown to contribute the highest level of displacements as shown in Movie S1 and S2.

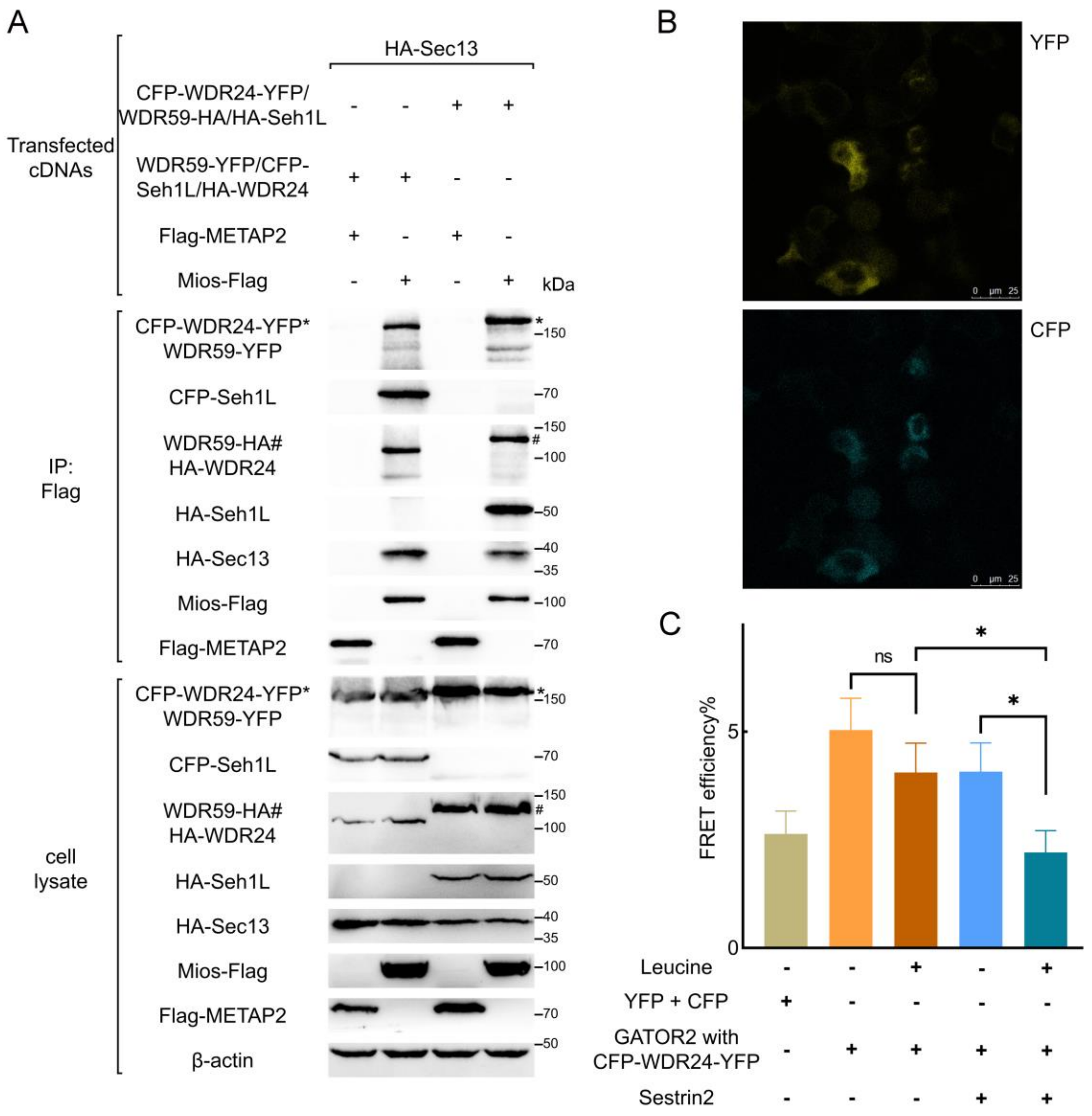

Figure S7. Effects of leucine and Sestrin2 on the conformational changes of GATOR2. (**A**) The combined expression of CFP or YFP showing unaffected complex formation of GATOR2 with METAP2 used as a negative control. The results are representative of three independent experiments. (**B**) Representative pictures of HEK-293T cells expressing YFP- or CFP-conjugated proteins. (**C**) Assessment of FRET efficiency of GATOR2 in response to leucine. Error bars mean ± sd, n≥15. * = $p<0.05$, ** = $p<0.01$, *** = $p<0.001$, **** = $p<0.0001$.

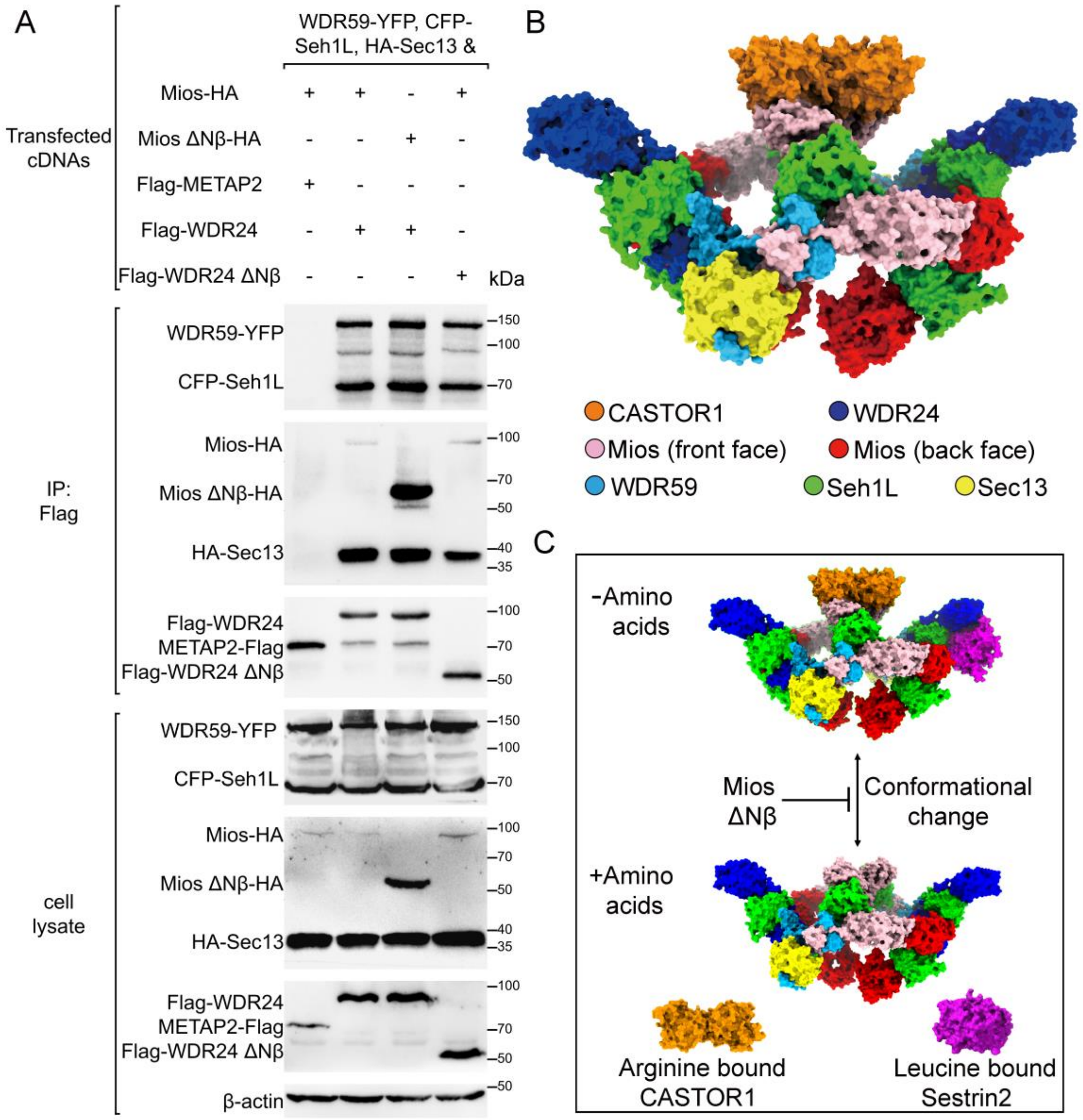

Figure S8. Effects of Mios-Nβ deletion on the conformational change in GATOR2. (**A**) Mios-Nβ deletion showing no effect on the GATOR2 integrity; WDR24-Nβ deletion partially impairs the formation of GATOR2 complex. The results are representative of three independent experiments. (**B**) Aligning AF2 model of CASTOR1-Mios Nβ with the architecture of GATOR2. Panorama view of CASTOR1-GATOR2 complex was visualized via USCF ChimeraX program. (**C**) A graphic model for the conformational change of GATOR2 in response to amino acids.
