# Supplementary material for: New insights into GATOR2-dependent interactions and its conformational changes in amino acid sensing
Source: Biosci Rep. 2024 Mar 13;44(3):BSR20240038. doi: 10.1042/BSR20240038 (PMC10938194; doi:10.1042/BSR20240038)

Movie S1 and S2. The 7th normal mode of GATOR2 generated from the output of online NMA server with two different views.

The orginal scans of the Western blots

Figure 2C: HA (IP)

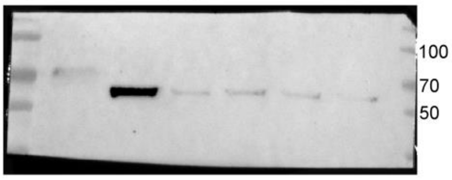

Figure 2C: Flag-WDR24 (IP)

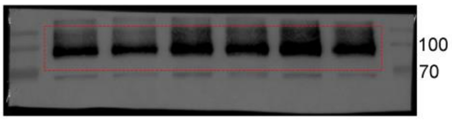

Figure 2C: METAP2-HA (cell lysate: lane 1)
HA-Sestrin2 (cell lysate: lane 2-6)

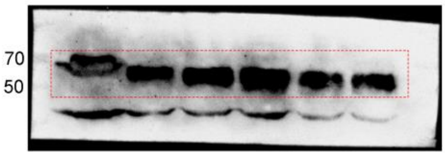

Figure 2C: Flag-WDR24 (cell lysate)

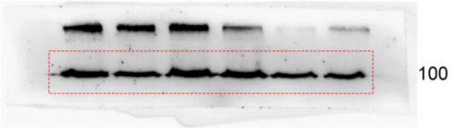

Figure 2C: β-actin (cell lysate)

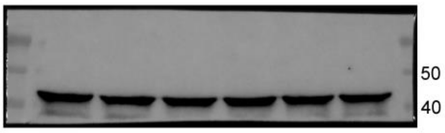

Figure 2D: HA-Sestrin2 (IP)

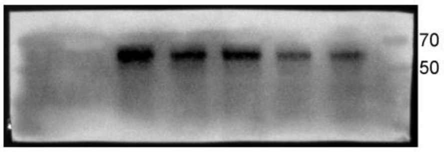

Figure 2D: Flag-METAP2 (IP: lane 1)
Flag-WDR24 (IP: lane 2-6)

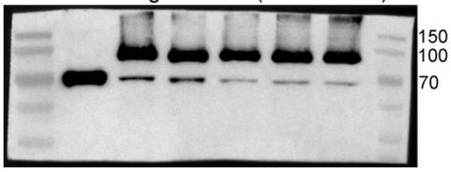

Figure 2D: HA-Sestrin2 (cell lysate)

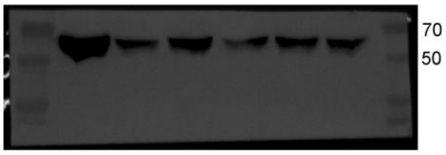

Figure 2D: Flag-METAP2 (cell lysate: lane 1)
Flag-WDR24 (cell lysate: lane 2-6)

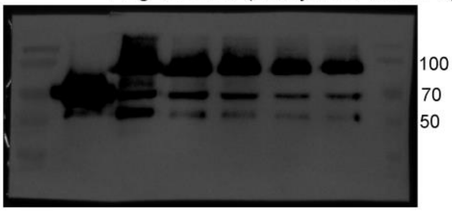

Figure 2D: β-actin (cell lysate)

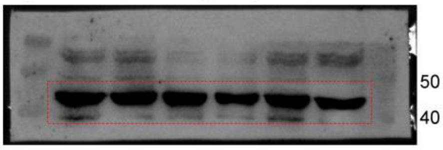

Figure 3A: P-T389-S6K1 (IP)

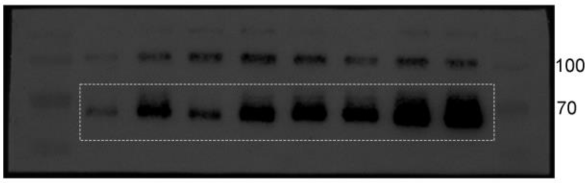

Figure 3A: Flag-S6K1 (IP)

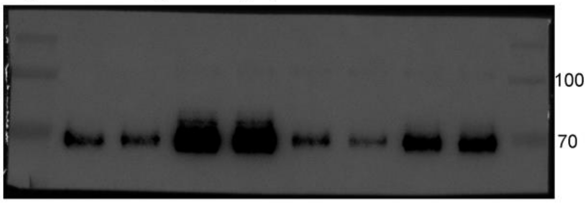

Figure 3A: METAP2-HA (cell lysate: lane 1-2)
HA-Sestrin2 (cell lysate: lane 3-8)

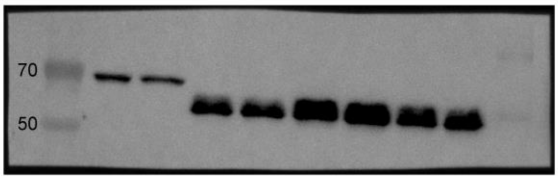

Figure 3A: mTOR (cell lysate)

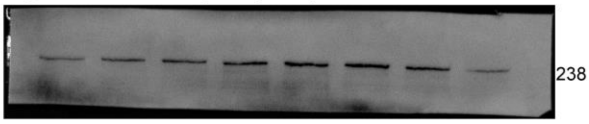

Figure 4D: HA-CASTOR1 (IP)

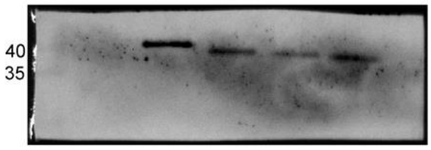

Figure 4D: Mios-Flag (IP)

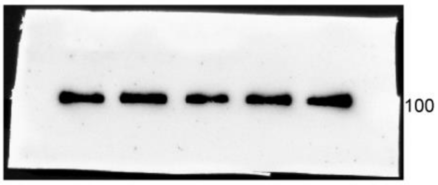

Figure 4D: METAP2-HA (cell lysate: lane 1)
HA-CASTOR1 (cell lysate: lane 2-5)

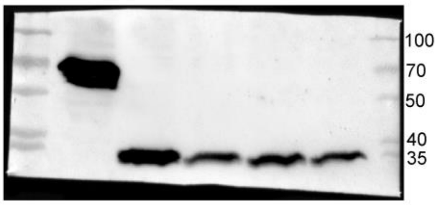

Figure 4D: β-actin (cell lysate)

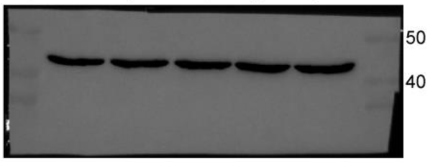

Figure 4E: Mios-HA (IP)

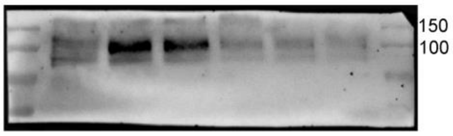

Figure 4E: Flag-CASTOR1 (IP)

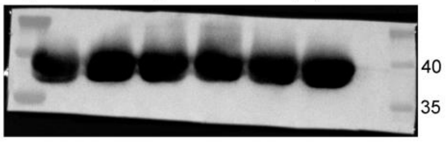

Figure 4E: METAP2-HA (cell lysate: lane 1)
Mios-HA (cell lysate: lane 2-6)

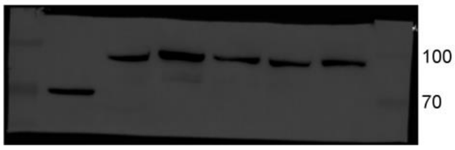

Figure 4E: Flag-CASTOR1 (cell lysate)

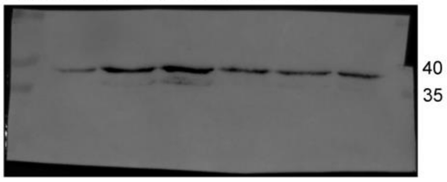

Figure 4E: β-actin (cell lysate)

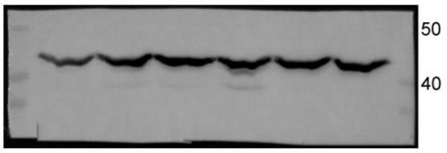

Figure S7A: CFP/YFP (IP)

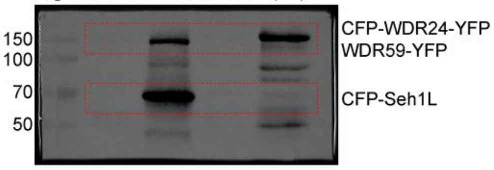

Figure S7A: HA (IP)

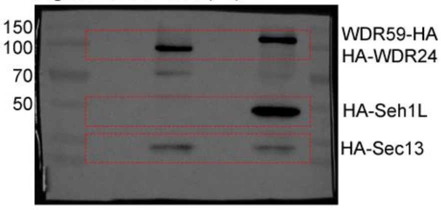

Figure S7A: Flag (IP)

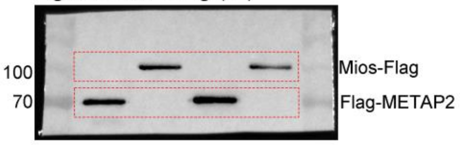

Figure S7A: CFP/YFP (cell lysate)

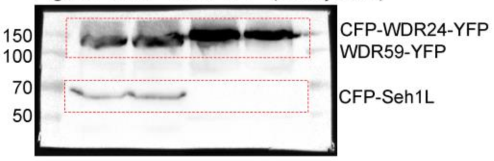

Figure S7A: HA (cell lysate)

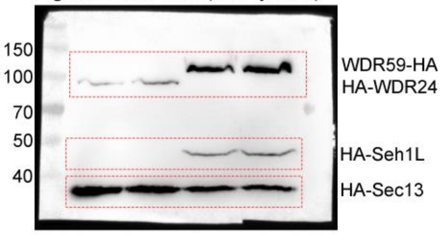

Figure S7A: Flag-METAP2 (cell lysate)

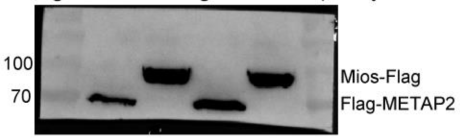

Figure S7A: β-actin (cell lysate)

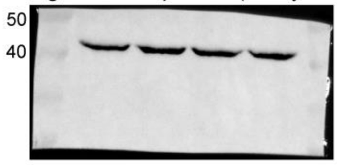

Figure S8A: CFP/YFP (IP)

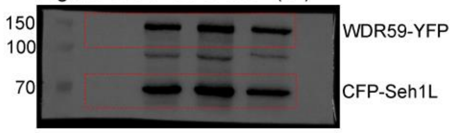

Figure S8A: HA (IP)

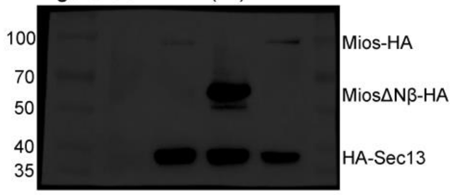

Figure S8A: Flag (IP)

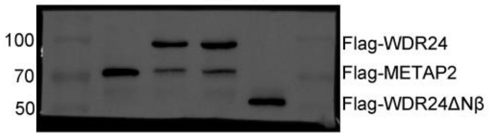

Figure S8A: CFP/YFP (cell lysate)

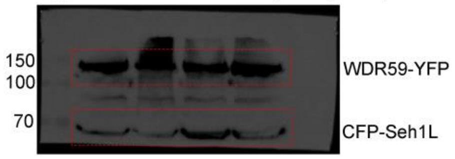

Figure S8A: HA (cell lysate)

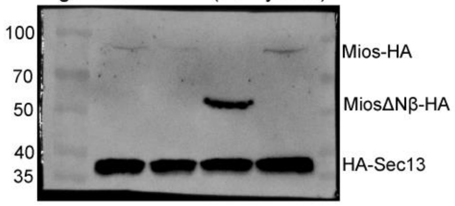

Figure S8A: Flag (cell lysate)

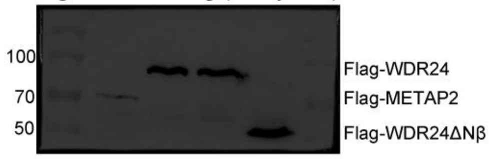

Figure S8A: β-actin (cell lysate)

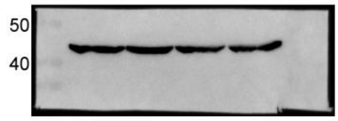

Supplement: Supplementary Movies S1 and S2 [file BSR-2024-0038_supp1.zip › Supplementary_Movie.pdf]
