# Supplementary figures and images for: New insights into GATOR2-dependent interactions and its conformational changes in amino acid sensing
Source: Biosci Rep. 2024 Mar 13;44(3):BSR20240038. doi: 10.1042/BSR20240038 (PMC10938194; doi:10.1042/BSR20240038)

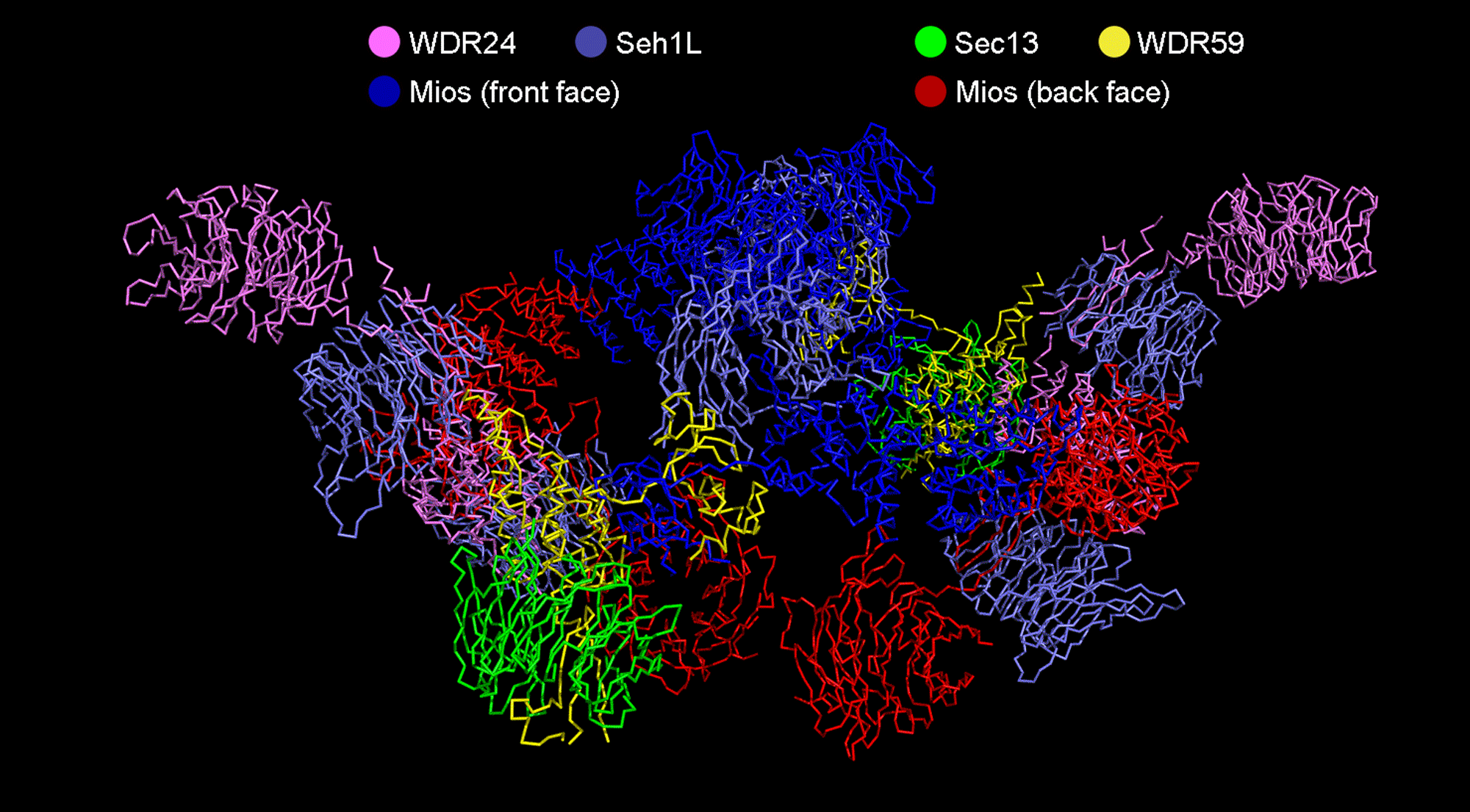

Supplement: Supplementary Movies S1 and S2 [file BSR-2024-0038_supp1.zip › BSR-2024-0038_suppS1.gif]

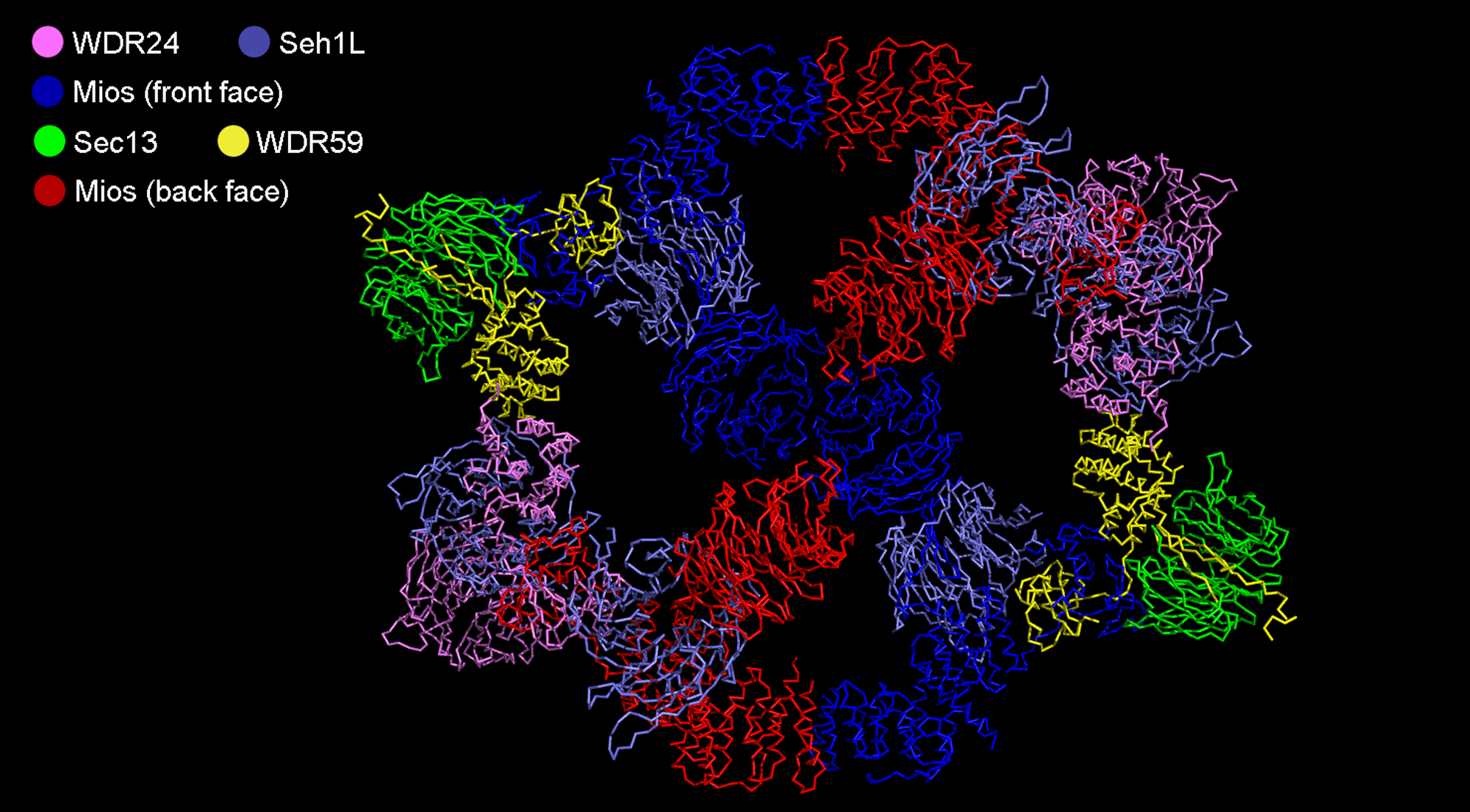

Supplement: Supplementary Movies S1 and S2 [file BSR-2024-0038_supp1.zip › BSR-2024-0038_suppS2.gif]
